# Role of phosphorus as micro alloying element and its effect on corrosion characteristics of steel rebars in concrete environment

**DOI:** 10.1038/s41598-022-16654-w

**Published:** 2022-07-21

**Authors:** Raja Rizwan Hussain, Abdulrahman Alhozaimy, Abdulaziz Al-Negheimish, D. D. N. Singh

**Affiliations:** 1 Center of Excellence for Concrete Research and Testing (CoE-CRT), Civil Engineering Department, College of Engineering, King Saud University, PO Box: 800, Riyadh, 11421 Saudi Arabia; 2 Civil Engineering Department and The Center of Excellence for Concrete Research and Testing, College of Engineering, King Saud University, Riyadh, Saudi Arabia; 3 Civil Engineering Department and Executive Director, Center of Excellence for Concrete Research and Testing, College of Engineering, King Saud University, Riyadh, Saudi Arabia; 4 Corrosion and Surface Engineering CSIR, National Metallurgical Laboratory, Jamshedpur and Currently R&D Consultant, IGNCA, New Delhi, 110001 India

**Keywords:** Chemistry, Engineering, Materials science, Nanoscience and technology

## Abstract

This communication reports the effect of phosphorus (P) added in micro concentration range in steel on kinetics, mechanism and growth of passive film in contact of chloride contaminated concrete. Electrochemical impedance spectroscopy, direct-current polarization, mass loss and Raman spectroscopic techniques were used to arrive at the findings. The results showed that an intentional addition of P in steel (0.064%) makes it more prone to uniform and localized corrosion (about 1.1 and 1.7 times) than the steel having low phosphorus (< 0.016%, present as tramp element) exposed under wet/dry conditions in simulated pore solution added with chloride and in the absence of this ion. A similar effect is also noted for the rebars embedded in mortars. Identification of corrosion products formed on steel rebars surface by Raman spectroscopy reveals thermodynamically stable maghemite and goethite phases on the surface of low P content steel. Unstable phase of lepidocrocite is recorded on the surface of higher phosphorus steel rebars. The findings are discussed with experimental evidence and taking clues from the published literature to arrive at plausible mechanism for this behaviour.

## Introduction

Many metallic and non-metallic elements namely carbon, sulphur, manganese, copper, vanadium, niobium, phosphorus etc. are added in steels in micro concentration ranges to achieve improvement in their properties. Literature survey reveals that researchers in the past had found that the added elements either improved^1,2^ or deteriorated^3–5^ the properties of steels. Very limited information on their role in changing the corrosion characteristics especially in concrete environments of the resultant micro alloyed steels are available in the literature^6–8^. It is more so for phosphorus added in steels. This element segregates in grain boundaries of steels causing brittleness and adversely affecting the fracture toughness^3–5^. In view of this the P content in steels is maintained at minimum level by some international standards^9^. Rebars embedded in concrete experience static and dynamic loadings during their service life. Some international standards for steels used to roll rebars therefore limit the maximum P content in chemistry of such steels. When weldability and enhanced ductility is required, rebars meeting ASTM A706^9^ standard is specified. ASTM A706 limits the phosphorus content to 0.035%. On the other hand, in USA and many other countries, ASTM A615^10^ is widely used and deals with the rebars for concrete reinforcement with no limitation on phosphorus content. The present research focuses on this type of rebars which has a much larger usage in the concrete construction industry.

It is known that the higher content of P in steel improves the atmospheric corrosion resistance of structures fabricated from such steels^1,2^. Some rebar manufacturers in certain countries expecting the same effect add extra phosphorus in steels used to roll the rebars. The rebars rolled from the scrap steels also contain higher content of phosphorus. The de-phosphorization of scrap steels is an expensive process and difficult to achieve the acceptable limit of this element. In view of the above facts, it is important to know the effect of content of P in rebars on their resistance to corrosion exposed in chloride contaminated concrete environments. Literature search reveals that extra addition of P in steels generally have a deteriorating effect on their resistance to corrosion exposed in environments with high humidity and water content. Kim et al.^11^ reported an adverse effect of P alloying in mild steel on its corrosion resistance in a gas desulphurisation system and attributed it to the increased hydrogen evolution reaction. A similar effect was also reported by Uhlig^12^ and Cleary and Greene^13^. Windisch et al.^14^ found an adverse effect of this element added in steel and tested in a calcium nitrate solution. The authors attributed it to the destabilising effect of phosphate (generated by the ionisation of P from the corroding steel) on the semi-protective Fe_3_O_4_ film. Krautschick et al.^15^ reported the accelerating effect of P and attributed it to the formation of a negatively charged species P^δ-^ that accelerated the attack. P has exhibited an augmenting effect on the stress corrosion cracking of steels in different test media^16–22^.

Balma et al.^23^ on the other hand reported that higher content of phosphorus in steel rebars had no effect on their resistance to corrosion in concrete environments. These results indicate that the protective or deleterious effect of P in steel is determined by the nature of the environment, the pH, and the anionic and cationic components surrounding the steels. Considering the aforementioned findings and the fact that P in steels segregates at grain boundaries, which may have a distressing effect on the soundness of the embedded reinforcement bars, it was prudent to test whether extra addition of this element in steels affects the performance of rebars exposed in concrete environments. This communication incorporates the results of tests of two types of rebars rolled from microalloyed C-Mn steel: one with the practically negligible content of phosphorus (present as the tramp element) and the other one having an intentionally added higher P content produced by some reinforcement bar manufacturers and allowed by many international standards^10,24,25^.

## Experimental methods and materials

### Test materials

Two types of steels one with a high P content (HP) intentionally added by the rebar manufacturer and the other with low P content (LP) having the chemical composition presented in Table 1 were used in this investigation. It is seen from the table that except phosphorus, all the elements are comparable in both the studied rebars. Phosphorus is unwanted in steels (unless added intentionally to get specific properties for a steel component). There was no extra addition of P in LP steel rebar. The P content of 0.016% came from the ore and other materials used to prepare the steel in mills. This content of P is known as the tramp element and remains in steels after de-phosphorization and exerts little adverse effects in the properties of the fabricated structures. The content of this element is considerably lower in LP (< 0.016%) than the HP (0.064%) steel rebar as shown in Table 1. The higher content P (0.064%) in HP steel bar is due to the extra addition of phosphorus as ferrophos during the process of steel making.

**Table 1:** Chemical analysis results for steel rebars obtained using optical emission spectrometer.

Steel rebar	Elements (wt.%)								
	C	Si	Mn	P	S	Cr	Ni	Cu	Fe
HP	0.13	0.20	0.98	0.064	0.013	0.035	0.014	0.39	Balance
LP	0.13	0.19	1.18	≤ 0.016	0.010	0.030	0.016	0.43	Balance

Both HP and LP steels were taken in the form of rebars of 16 mm diameter. After hot rolling, both the rebars were subjected to quenching and tempering treatments. Approximately 10–12% of their outer diameter was transformed into a tempered martensite rim. Appropriate lengths of the test specimens were cut by a diamond cutting machine from a single full-length rebar avoiding localized heating during the cutting.

### Test environments

#### Simulated pore solution saturated with lime (SPSL)

The SPSL was made as detailed in our previous publication^8^. This involved mixing of 0.55 M KOH, 0.16 M NaOH, and 0.017 M Ca(OH)_2_ in distilled water, stirring on magnetic stirrer for two hours followed by filtration on filter paper to remove insoluble lime. The solution was stored in a high-density polyethylene (HDPE) plastic airtight container. The pH of the solution was 13.5.To assess the effect of chloride on the performance of rebars, 0.6 m chloride ion was added in the form of sodium chloride in SPSL.

#### Mortars

**Figure 1:**
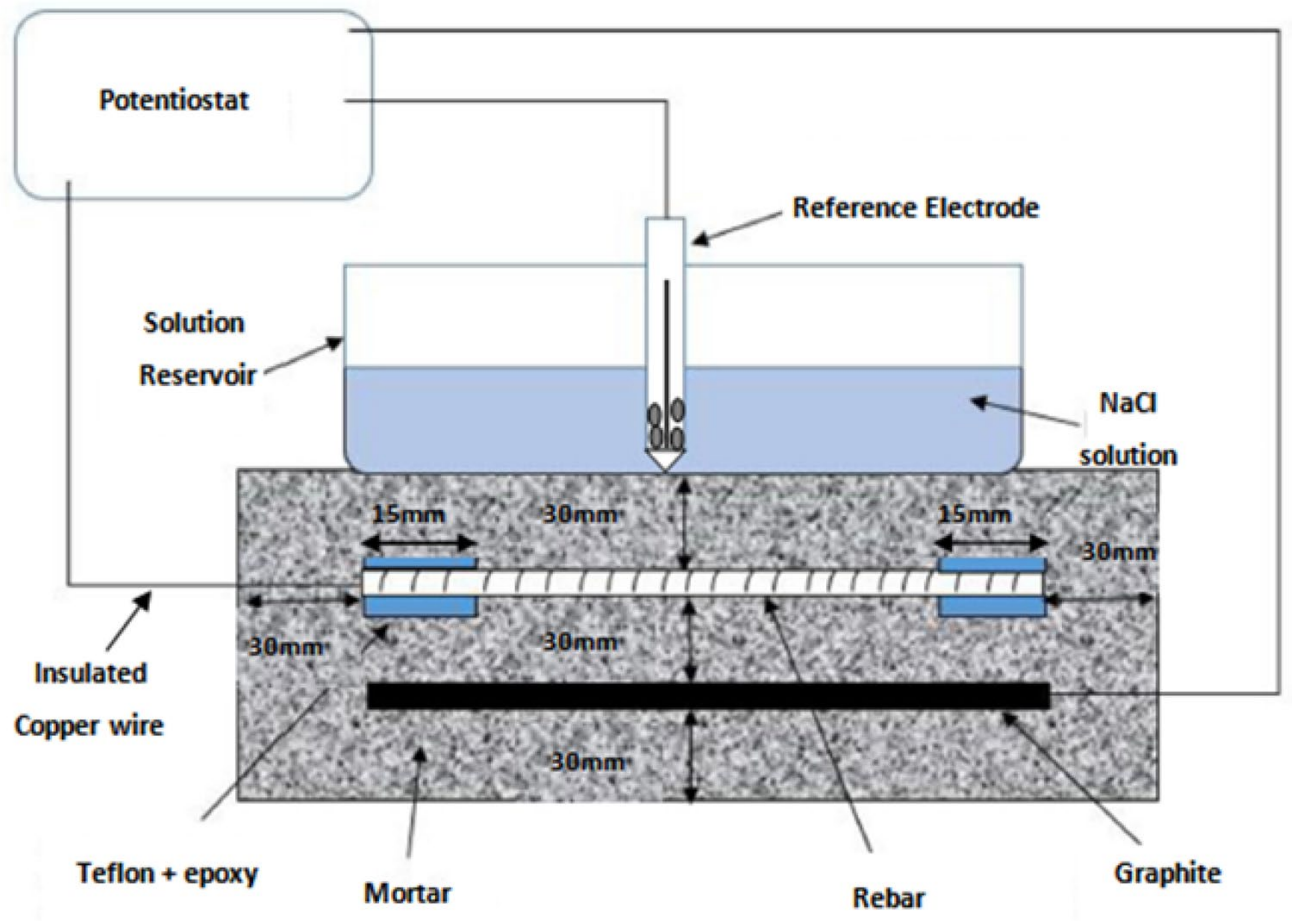
Schematic diagram showing the geometry of mortars used to evaluate the proneness to corrosion of HP and LP rebars.

The geometry, composition and method of preparation of mortars, size of embedded rebars and graphite rods were the same as described in authors’ previously published papers^7,8,26^ and schematically depicted in Fig. 1. The embedded rebars and graphite rods respectively were used as working and auxiliary electrodes during the electrochemical studies. The two ends of rebars and graphite rods (their 15 mm lengths) were coated with epoxy and Teflon tape to avoid crevice corrosion. Copper wire was soldered on the surface of rebars and tightly wrapped on graphite rods before the application of Teflon and epoxy coating. The other end of the wires coming out from the mortars was used to take electrical contacts. As seen from the Fig. 1 out of 150 mm length of the rebar only 120 mm was open to exposure in the mortar. A 30 mm cover thickness of mortar was available for the steel bars from all the casting sides. The rebars’ surface was abraded on motorized abrading wheel to remove loose rust and scales followed by their de-oiling in acetone.

Three sets of mortars were prepared, each embedded with “HP” and “LP” rebars, as prescribed in ASTM –C192. After 24 h of casting the mortars were de-molded and cured for 28 days in a humidity chamber maintained at 95% RH and a 25 °C temperature.

### Test methods

#### Wet /dry exposure in SPSL

These experiments were performed as described in the author’s past research^27^. This involved cutting of 20 mm length of rebars samples, mounting them in acid/alkali resistant resins leaving one end of their cross sectional area (2 cm^2^) to be exposed in the test electrolytes. The cross sectional area of these samples was mirror polished. After swabbing with acetone and drying, the polished specimens were weighed on an electronic balance with an accuracy of 0.0001 g. Six specimens of each type of reinforcement bar were prepared. Subsequently, two drops of the test electrolyte with the aforementioned compositions were placed on each surface (for each type of rebar, SPSL was dropped onto three specimens and chloride added SPSL (0.6 M chloride ions added as sodium chloride) was dropped onto the other three). This quantity of solution was enough to wet the bare cross section area of the test specimens. These specimens were then placed in a chamber for 78 h, where the relative humidity and temperature were controlled at 95% and 45 °C, respectively. After this period of exposure, the specimens were removed from the chamber. Then, two drops of the test electrolytes were again dropped onto the specimens, and they were returned to the chamber for another period of 78 h. After 156 h of exposure ((wet cycle), the specimens were removed from the chamber and kept in the laboratory environment for 156 h, where the temperature varied in the range of 25 ± 5 °C (dry cycle). This process of 312 h (13 days) was taken as one cycle. After the completion of each cycle, the aforementioned procedure of applying the test electrolyte, drying etc. was continued for seven cycles. After the completion of seven cycles (total of 91 days), the rust on the surface of the tested specimens were cleaned in Clarke’s solution as detailed in ASTM G1-90 (re-approved 2010)^28^, and the corrosion losses were computed using the average weight-loss data of the three samples of each steel.

#### Electrochemical tests in simulated pore solutions

##### Potential-time measurement

**Figure 2:**
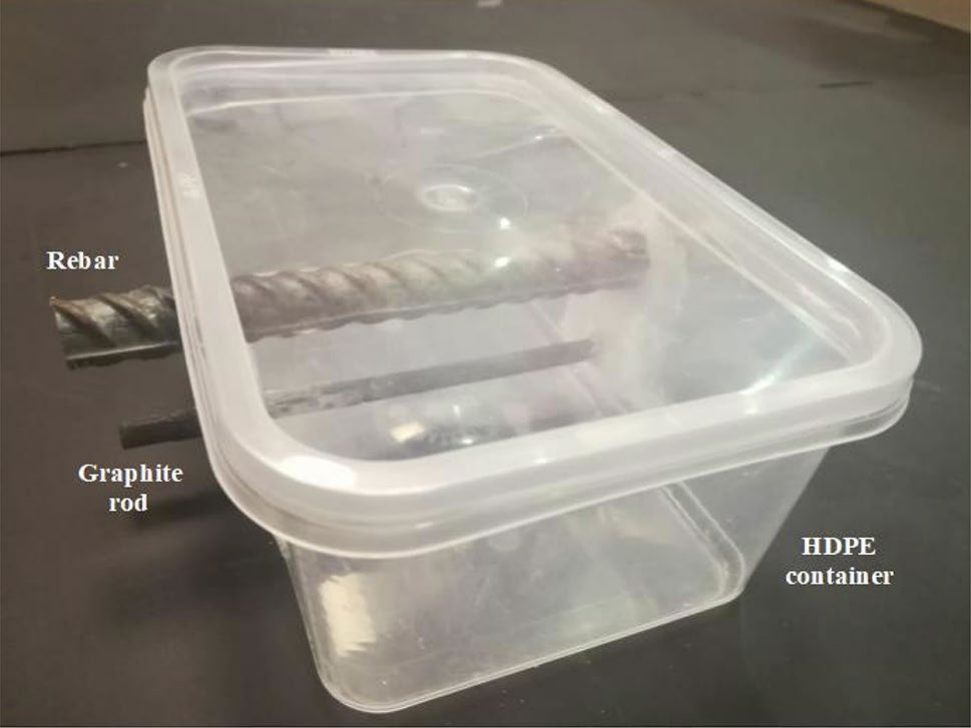
Photograph of the cell used to expose the rebars test samples in simulated pore solutions to conduct the electrochemical experiments.

Potential–time measurement tests were performed by exposing the appropriate area of samples in the test environments after de-scaling, abrading, and emery polishing of their surfaces. For this test each rebar 15 cm length were fitted in electrochemical cells shown in Fig. 2. A graphite rod 16 mm diameter of the same length as the rebar was fitted horizontally 20 mm above the rebars. This rod was used as the auxiliary electrode in the cell during the DC polarization and electrochemical impedance studies as described in next sections. The two ends of the graphite rods and rebars coming out from the test cell were blocked with epoxy resin to make them impervious to liquid diffusion. To avoid crevice corrosion 0.5 cm length of rebar coming in contact of the test electrolyte in the test cells at both ends were first blocked with Teflon tape wrapping around the rebar followed by covering this part with epoxy resin. 10 cm length of the rebars with active area of 50.25 cm^2^ was exposed in the test electrolyte. A calomel electrode fitted with Luggin capillary was positioned very close to the surface of the rebar samples. There after SPSL (without chloride addition) and SPSL added with chloride ions (0.6 M) was poured in three sets each of the test samples. The change in open circuit potential with time was measured connecting the wire leads of rebar samples and Calomel electrode with a potentiostat.

##### Cyclic anodic polarization studies

To assess the severity of uniform and localized corrosion of the two rebars exposed in chloride added and chloride free SPSL test electrolyte, the cyclic anodic polarization experiments were performed in the same cell (Fig. 2) and solutions after completing the potential-time experiments. The scan rate of potential for both the forward and backward scan was 0.1 mV/s. Maximum anodic current was fixed at 10 mA/cm^2^.The data were analysed by using a DC 105 software of M/S gamry instrument. Three sets of experiments were performed for each steel rebars and the two data close to each other were averaged and produced in the paper. The experiments were performed at 25 ± 1 °C.

##### Electrochemical impedance studies (EIS) in pore solutions

These experiments were conducted in the similar cells as detailed in Fig. 2. However, the new samples and electrolytes were taken for these studies. Like polarization experiments, in this case also three sets of experiments were performed for LP and HP steel rebars. The tests were performed after different intervals of exposure of the samples by applying a 10 mV sinusoidal voltage at the open-circuit potential of the working electrode while changing its frequency from100 kHz to 0.01 Hz. The EIS data were analysed using CMS 300 Software of M/S Gamry instruments.

##### Electrochemical impedance studies (EIS) of rebars embedded in mortars

The EIS tests of rebars embedded in mortars were performed under wet condition of the mortars as described in our earlier publication^29^ .The prepared mortars as described in “Mortars” section were exposed for 10 days in a 3.5% sodium chloride solution, followed by drying in laboratory environment for 20 days. This period of wet/dry treatment (30 days) was considered as one cycle. Cyclic wet-dry treatment of mortars is reported to have an accelerating effect on the onset and propagation of corrosion on the surface of the embedded rebars^30^.

##### Characterization of corrosion products on the surface of the tested rebars

Raman spectroscopy of the corrosion products collected from the test samples was performed as described in reference^31^. An Almega Dispersive micro-Raman spectroscope equipped with the beam of an Nd:YAG (Nd-doped yttrium aluminium garnet; Nd:Y_3_Al_5_O_12_) green laser with a wavelength of 532 nm was used. The laser was maintained at low power to avoid the transformation of corrosion products due to the heating effect. The locations of the specimens to be studied were focused through an Olympus microscope at a magnification of 50 × . The sample holder incorporated a motorised platform with a jockey to facilitate precise focusing and mapping at suitably specified portions of the specimens. The grating was 672 lines/mm, with a 25-µm pinhole. Prior to the analysis of the samples, the instrument was calibrated by using pure Si at the peak of 532 cm^−1^.

## Results

### Corrosion rate of steel bars under wet/dry treatments with SPSL

**Figure 3:**
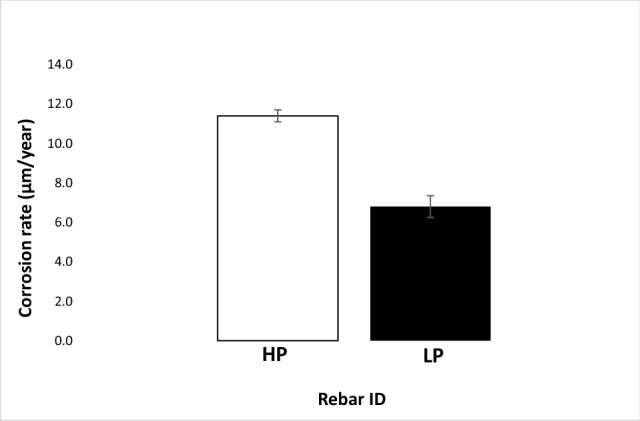
Corrosion rate of the HP and LP steel rebars in chloride free SPSL solution after seven cycles of wet/dry treatments.

**Figure 4:**
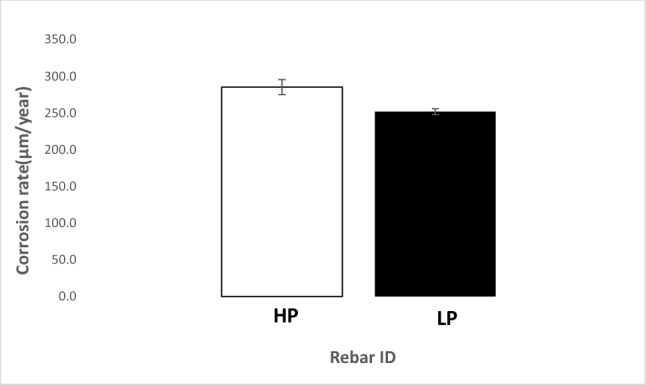
Corrosion rate of HP and LP steel rebars under the influence of wet/dry treatment using chloride added SPSL solution.

The corrosion rates of the two studied rebars based on mass loss after seven cycles of wet/dry treatments with SPSL (with and without chloride) were computed considering that uniform corrosion took place on their surfaces. The thickness losses of HP and LP rebars are recorded as histograms in Figs. 3 and 4. As shown in Fig. 3, which incorporates the losses under the influence of the SPSL electrolyte (free of chloride), the HP steel rebar experienced a significantly higher corrosion rate (by a factor of approximately 1.7) than the LP rebar. A significant rate of corrosion of the two steel (6.8 and 11.4 µm/year average values respectively for LP and HP rebars) especially in chloride free SPSL is surprising. Pourbaix Fe-H_2_O diagram predicts the formation of protective film of Fe_3_O_4_ on iron surface up to pH 14 (at 1 N NaOH i.e., 4% sodium hydroxide water solution). The simulated pore solution used in the present study is well below 1 N NaOH (0.55 M KOH + 0.16 M NaOH + 0.017 M Ca (OH)_2_ dissolved in 1 L of water) and pH of this solution was in the range of passive zone of the Pourbaix diagram (pH 13.5). The abnormally high rate of corrosion of the studied steel may be due to the leaching out of phosphorus from the studied steel rebars in the pore solution which facilitated the oxygen reduction reaction at the steel-test solution interface. Many organo-metallic P doped catalysts are reported to increase oxygen reduction reactions in alkaline solutions because of interaction of leached out P with alkali solutions^32–34^. P in alkaline solution disproportionate to phosphene (PH_3_) and sodium hydrogen phosphide (NaH_2_PO_4_)^35^. Phosphene is a known accelerator for the corrosion reactions^36^. Higher the leached out P at the interface (in case of HP) greater is the rate of corrosion.

The corrosion rate under the influence of the chloride-added pore solution was very high for both the steels as illustrated in Fig. 4. However, the difference in corrosion rate was marginal (under this test condition the HP steel rebar corroded approximately 1.1 times faster than the LP steel). These results suggest that the passive film formed on both the steels was susceptible to chloride-induced deterioration.

**Figure 5:**
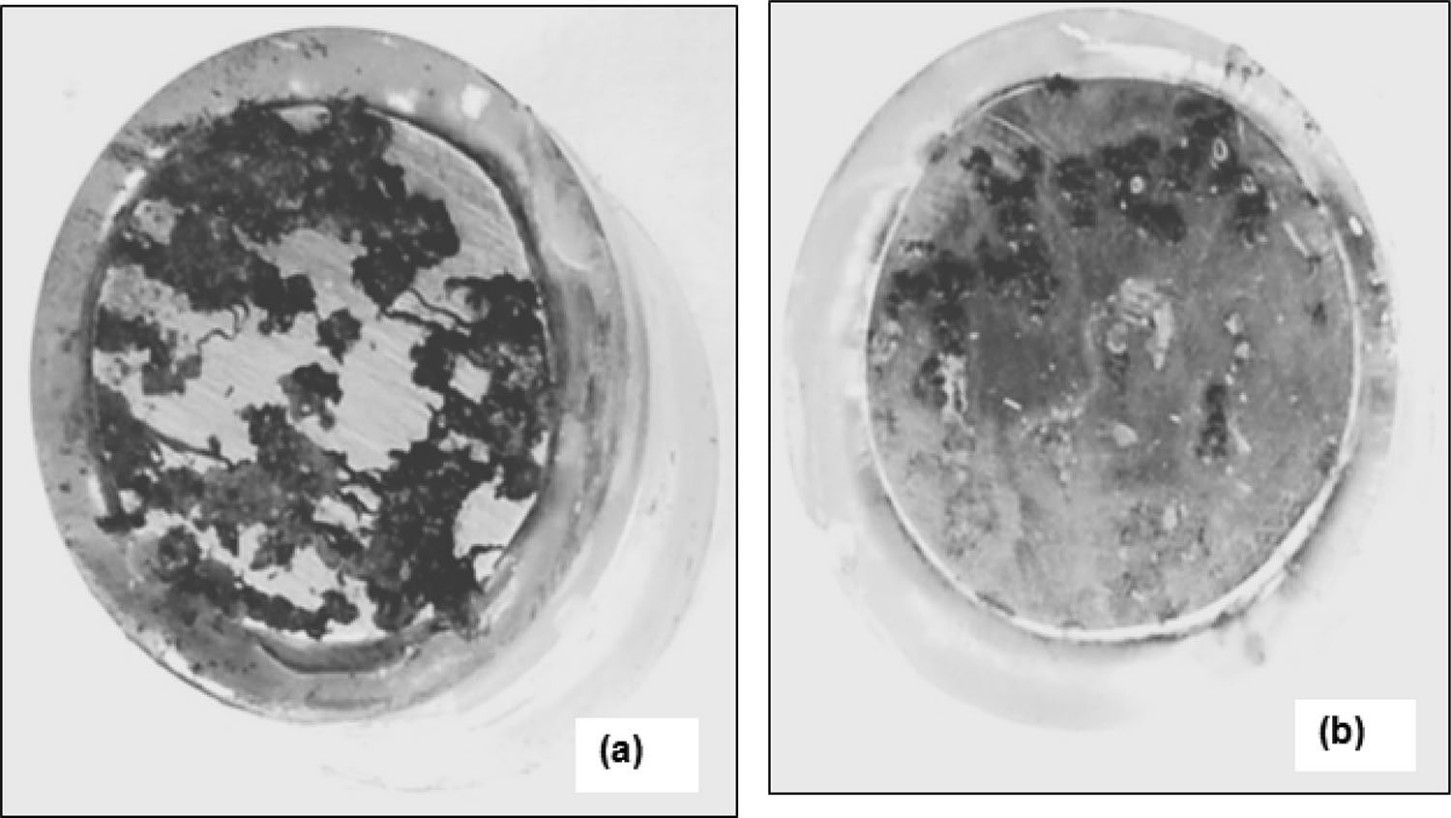
Digital photograph showing the accelerated localized attack on HP steel than LP rebars after their wet/dry treatments–wetting with chloride added SPSL (**a**) HP, (**b**) LP.

Digital images of the surfaces of the rebars after seven cycles of wet/dry treatments (wetted with chloride added SPSL), after cleaning the rusts as described previously, are shown in Fig. 5. The digital images very distinctly show that the HP rebars suffered with extensive localized corrosion attack than the LP rebars.

### Change in open circuit potential with exposure time

**Figure 6:**
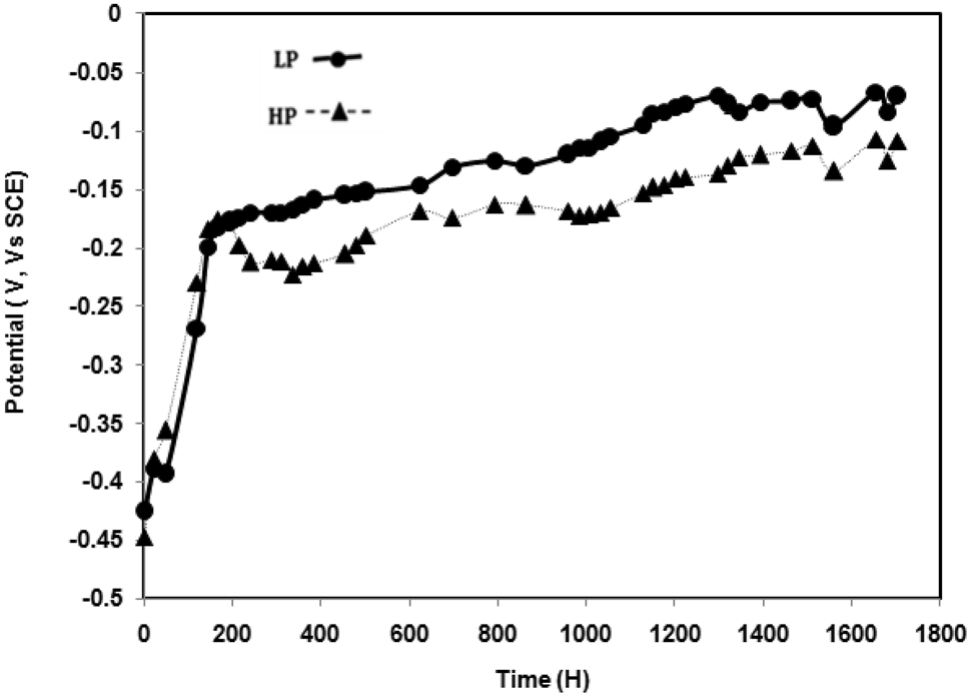
Change in corrosion potential with time for steel rebars exposed in chloride free SPSL.

**Figure 7:**
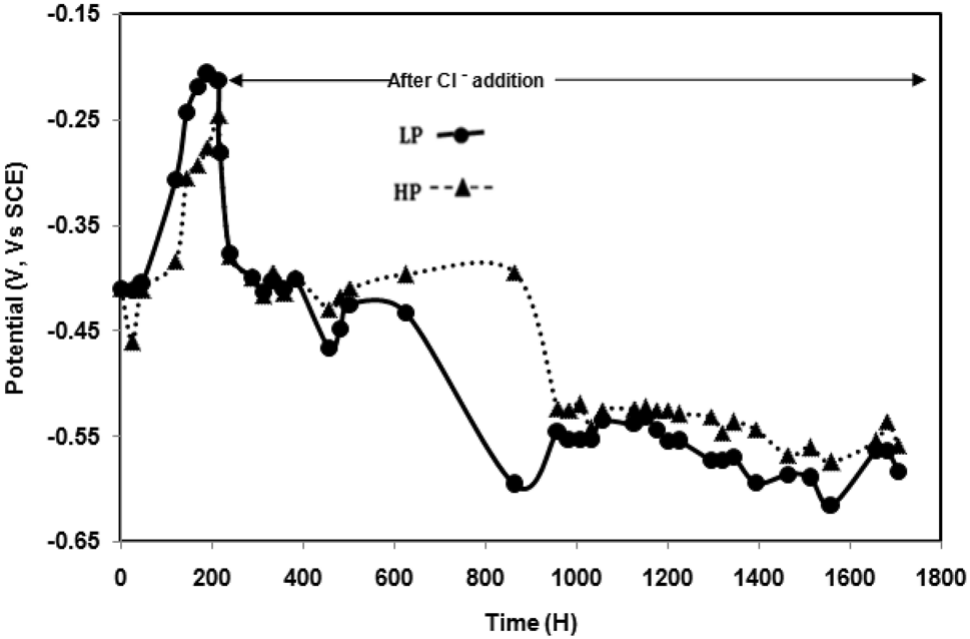
Change in corrosion potential with time for steel rebars exposed in chloride added SPSL.

Figure 6 shows the change in the open-circuit potentials of the two steels with the passage of exposure time in chloride free SPSL. The results show that the potentials of both the steels become nobler with the passage of time indicating the strengthening of the passive film on their surfaces. The changes are very sharp up to 200 h of exposure. Thereafter, the passive film on their surfaces stabilised, resulting in sluggish ennobling of the potentials. Although the difference is not very significant, the potential–time plot for the LP steel exhibits greater nobility than that for the HP steel at all the durations of exposure. In contrast, the potential variations for the two steel rebars significantly moved in the active direction after addition of chloride at 200 h of exposure (Fig. 7). These findings corroborate the results shown in Figs. 3 and 4, where the corrosion rate for the LP steel under the influence of chloride free SPSL was considerably lower than that for the HP steel (Fig. 3), whereas with the chloride-blended electrolyte, the difference was marginal (Fig. 4).

Chloride ion is a well-known activator for destabilization of passive film on steel surfaces exposed in most of the test solutions. Under a favourable condition this effect is more pronounced as currently noted for the HP steel rebars. The high negative charge and small ionic size of chloride ions enables them to easily adsorb onto and hydrolyze the passive film^37^.The ionic adsorption on oxidized metal surfaces depends on their pH_o_ (that is, the pH at which there is a zero net surface charge of the oxide layer, presented as:

1 $${\text{pH}}_{0} = \left[ {{\text{OH}}^{-} } \right]_{{{\text{surf}}}} + \left[ {{\text{H}}^{ + } } \right]_{{{\text{surf}}}}$$

This layer attains negative charge under a test condition where the pH value of the test electrolyte in vicinity of the passive metal exceeds than pH_0_.The pH_0_ value for iron oxide is 8.8^38^ suggesting that it should attain negative charge in alkaline pore solution and discourage the adsorption of chloride ions. The vulnerability of steels to pitting attack by chloride ions above a threshold concentration is a well-known phenomenon. This suggests that the mechanism of localized corrosion on steel surface in contact of pore solution needs to be looked from the other angles of mechanism. Different theories have been developed for the onset and propagation of pits on steels in the presence of chloride ions. Kruger and Ambrose^39^ opined that onset of pitting by chloride ions takes place after an induction period of exposure. During this induction period no changes in nature of the passive film occur. Mcbee and Kruger^40^, however, suggested that chloride ions affect the passive film before its breakdown. Adsorption of chloride ions onto a hydroxylated oxide surface is hypothesized by Szklarska-Smialowskaet al.^41^ as shown below:

2 $$MeO.H_{2} O \,\left( {hydrated\,passive\,film} \right) + Cl ^{ - } \to (MeO.H_{ \to HCl}^{ \to OH} )_{ads} + e^{ - }$$

where MeO denotes the passive film on steel surface. It is suggested that entry of electrons in passive film through the above proposed reaction increases the electron density in the film by dynamic equilibrium of adsorption and desorption. It is reported that an increase of surface electron density leads to a contractive surface stress which may destabilize the surface film^42^. This disruption in the film transformed the stable oxide phases of the film into unstable phases of the rust. The mechanism proposed by Szklarska-Smialowska et al.^41^ as proposed above appears operative for the currently studied HP and LP steel rebars. The values of capacitance (C_dl_) for the double layer formed at the corroding interface of LP and HP steel bars are found considerably higher for SPSL added with chloride than those for chloride free solutions (an indication of unstable film) (described latter in “Electrochemical impedance studies in pore solutions” section).

### Potentiodynamic cyclic polarization

**Figure 8:**
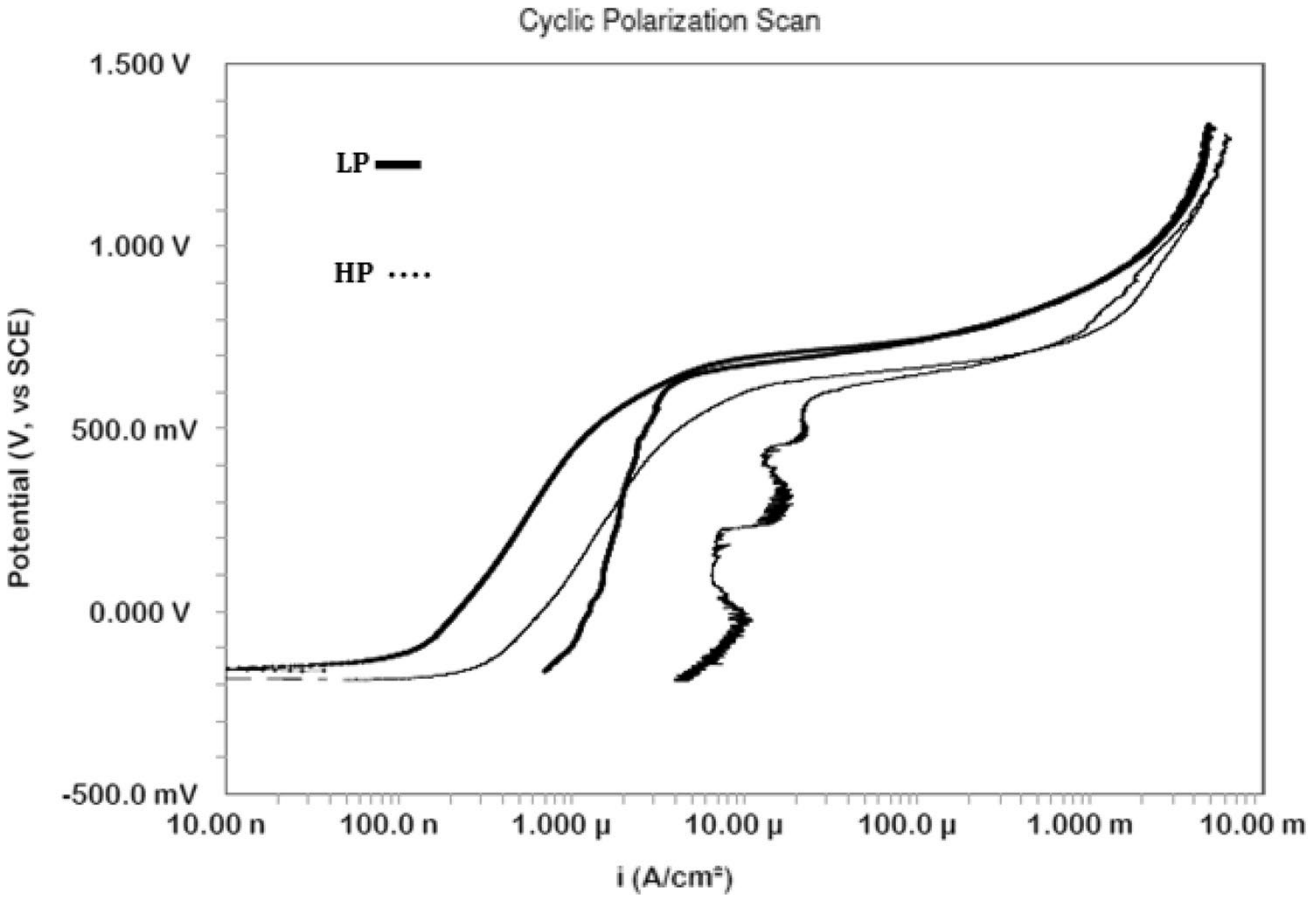
Cyclic polarisation of HP and LP steel bars after 1700 h of their exposure in chloride free SPSL.

**Figure 9:**
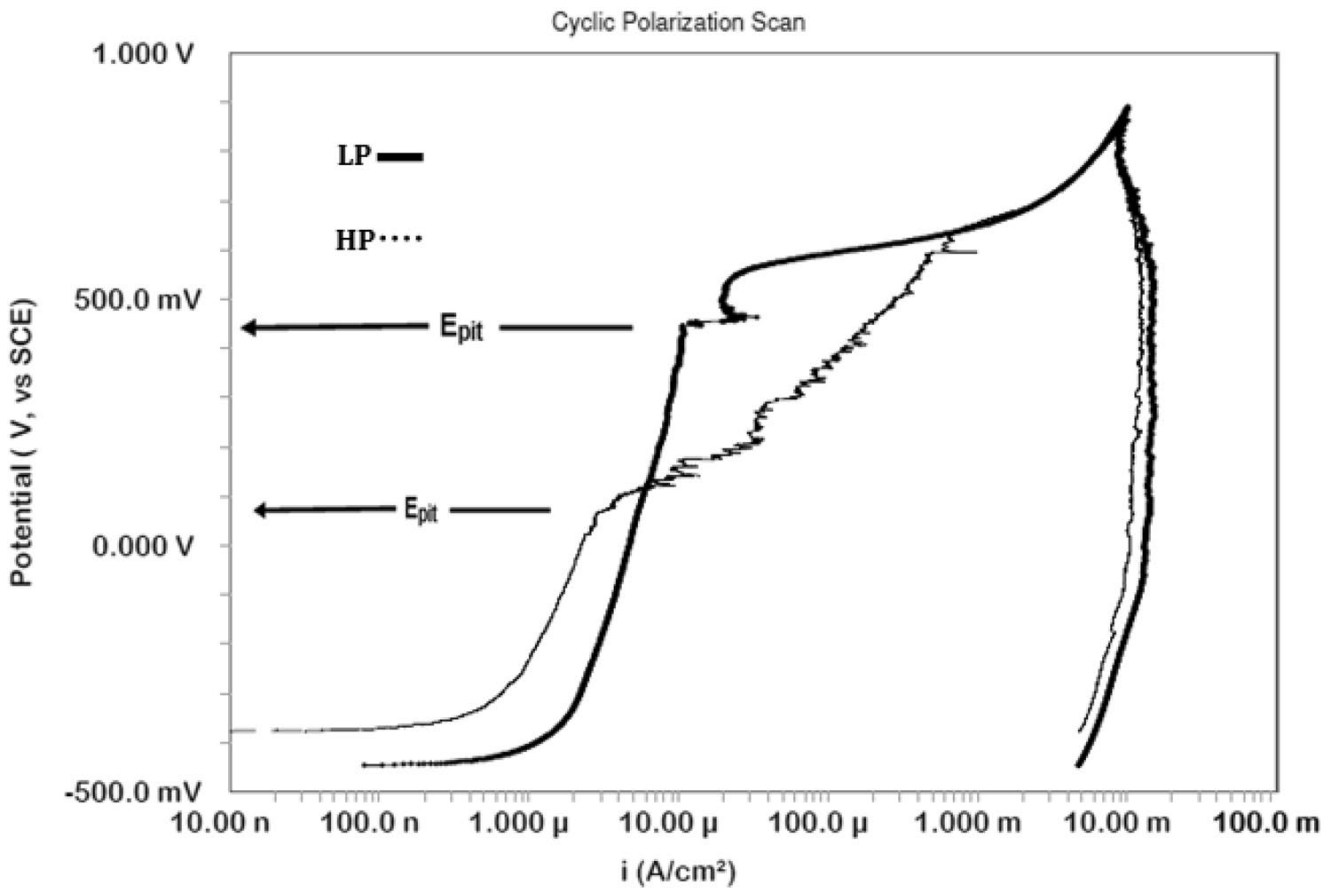
Cyclic polarisation of HP and LP steel bars after 1700 h of their exposure in chloride added SPSL.

The pitting and polarization behaviours of LP and HP steels were studied after 1700 h of their exposure in SPSL and chloride added SPSL. The corresponding plots are presented in Figs. 8 and 9, respectively. In the absence of chloride ions, a surge in current at around 0.6 V in the curves of both the steels is noted which appears as if an onset of pitting on the surface of steels occurred. This surge in current, however, is not due to pits initiation but onset of oxygen evolution reaction. For both the steels the back scanning of potential did not change the current and the curves retraced to the forward scan in the oxygen evolution region. However, it resulted in the formation of a positive current loop for both the steels during the back scan of the potential in the passivation region. It appears that during the forward scanning the passive film formed on the surface of both the studied steels deteriorated and the lowering of potential did not help in repairing the damaged film resulting in higher current density.

In chloride added SPSL, both the steels suffered pitting attacks at different anodic potentials below the oxygen evolution potential, as indicated in Fig. 9. In lower anodic potential region the HP steel rebar exhibits lower current density than the LP steel bar indicating that the film formed on the surface of the former steel rebar was more protective than the latter one. This observation is surprising in view of the fact that the rust accumulated on HP steel was substantially thicker and suffered greater metal loss than the SP rebar (Fig. 4). It appears that carbonate formed in SPSL (due to the reaction of alkaline solution with atmospheric carbon dioxide) got embedded in the pores of thick rust formed on HP rebars surface and blocked them. This probably resulted in its reduced dissolution rate. The protection against corrosion by rust layers has been reported by other researchers also^43^. Many other published data reported improved corrosion resistance of steel due to entangled carbonate crystals in rust^44,45^. Increased anodic potential however generated higher current density than the LP steel rebar above the pitting potential (indicated by Epit , Fig. 9). These results suggest that the carbonate blocked rust layer on HP rebar was more vulnerable to dissolution than that formed on LP steel rebar. The plot for the presence of chloride ions in the test electrolyte and a longer duration of exposure, the passive film on the rebars’ surface was not very stable. Due to poor and defective film formed on the steel’s surface it was vulnerable to the increased anodic potentials and complete breakdown of the passive film took place at different anodic potentials depending on their P content. The points marked as E_pit_ on the curves for LP and HP steel bars (Fig. 9) show that onset of pitting on LP and HP rebars took place respectively at 0.48 V and 0.05 V. The E_pit_ value for LP steel rebars recorded during this study is considerably nobler than that reported by Li and Sagali^46^. They reported values of E_pit_ ranging between − 0.3 V to + 0.1 V (with varying chloride content) for low phosphorus (0.007%) rebar having copper content very close to the steel used in this study (0.37%). This is probably due to insufficient time given by the authors of reference^46^ to stabilize the passive film in SPSL (3 h). In our case the time allowed before starting of the cyclic polarization tests was 1700 h which is good enough to form stable passive film on the surface of exposed rebars^29^. It is further noted from the curves of Fig. 9 that both the steels generated a large positive current loop, indicating that once the pits were formed on their surfaces, back scanning the potential in the active direction did not help the re-passivation of the generated pits (Fig. 9).

### Electrochemical impedance studies in pore solutions

**Figure 10:**
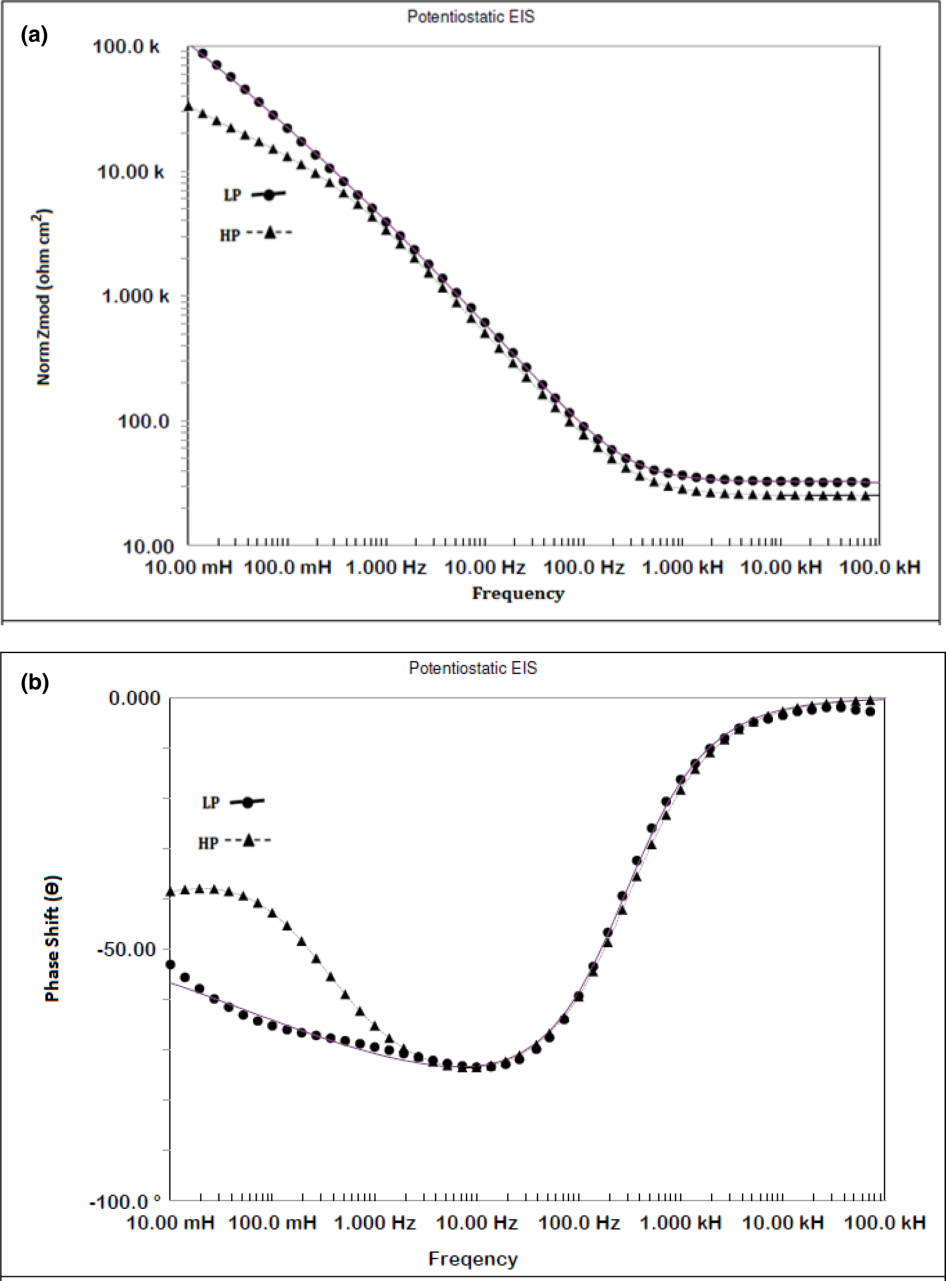
(**a**) Electrochemical impedance Bode plots for HP and LP steel bars after 24 h of exposure in chloride free SPSL. (**b**) Electrochemical frequency–phase shift Bode plots for HP and LP steel bars after 24 h of exposure in chloride free SPSL.

**Figure 11:**
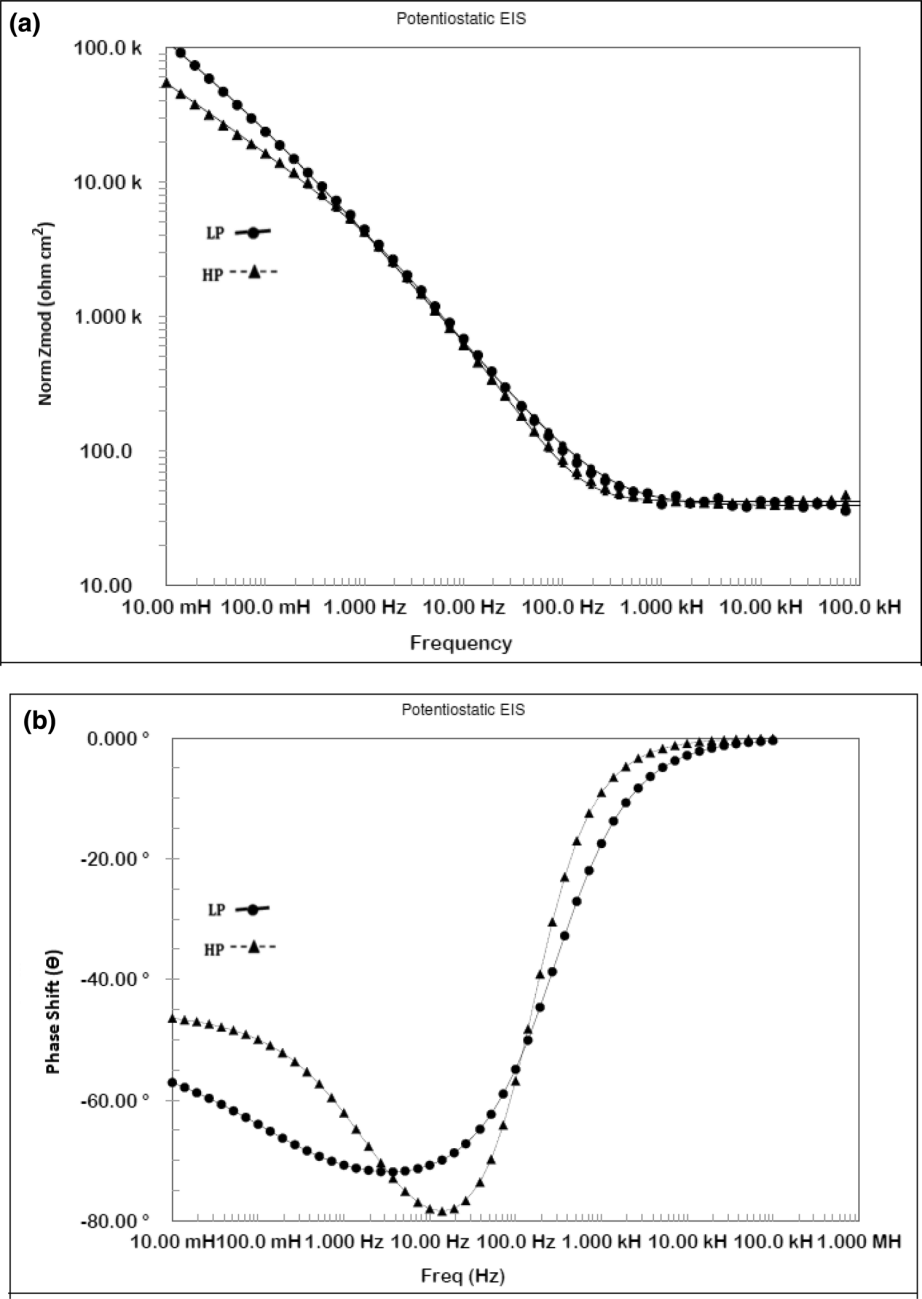
(**a**) Electrochemical frequency–impedance Bode plots for HP and LP steel bars after 192 h of their exposure in chloride free SPSL. (**b**) Electrochemical frequency–phase shift Bode plots for HP and LP steel bars after 192 h of their exposure in chloride free SPSL.

Potentiostatic electrochemical impedance plots for the two steels after 24 h of their exposure in chloride free SPSL are shown as Bode plots in Fig. 10a,b. The plots of Fig. 10a show that at the lowest studied frequency (10 mHz), which provides the value of maximum impedance (Z_max_) for a corroding interface is greater for the LP than for the HP steel rebars, indicating that the passive film formed in 24 h on the surface of the LP rebar was more protective than that on the HP rebar. The log frequency–phase shift plots for the two steels shown in Fig. 10b exhibit different features. While the HP steel exhibits two maxima—one in the frequency range of 10 to100 Hz and the other around 10 to 100 mHz—the LP steel appears to have a single maximum at around 10 to 100 Hz. The lower frequency maxima are not visible in this case. The low and intermediate frequency maxima for the HP steel are attributed to the corrosion reactions occurring at the metal–solution interface and the passive film on the steel surface, respectively. The same samples kept exposed for 192 h was again subjected to EIS tests to study the effect of the exposure time on the nature of the passive film formed on the surface of the two steel . The frequency–impedance and frequency–phase plots shown in Fig. 11a, b for this duration of exposure indicate that the trend was the same as noted for shorter duration of exposure (Fig. 10) and the impedance ((Z_max_) corresponding to the lowest studied frequency (10 mHz) was greater for LP than the HP steel rebar (Fig. 11a). Under this exposure duration, the impedance ((Z_max_) corresponding to the lowest studied frequency (10 mHz) was greater (Fig. 11a) than that for the shorter exposure duration (24 h, Fig. 10a). Further, both the steels though apparently exhibit a single maxima at approximately in the range of 10 to 100 Hz (Fig. 11b) but fitting of the data was satisfactory only after the Warburg diffusion element in the corroding electrical circuit was added.

**Figure 12:**
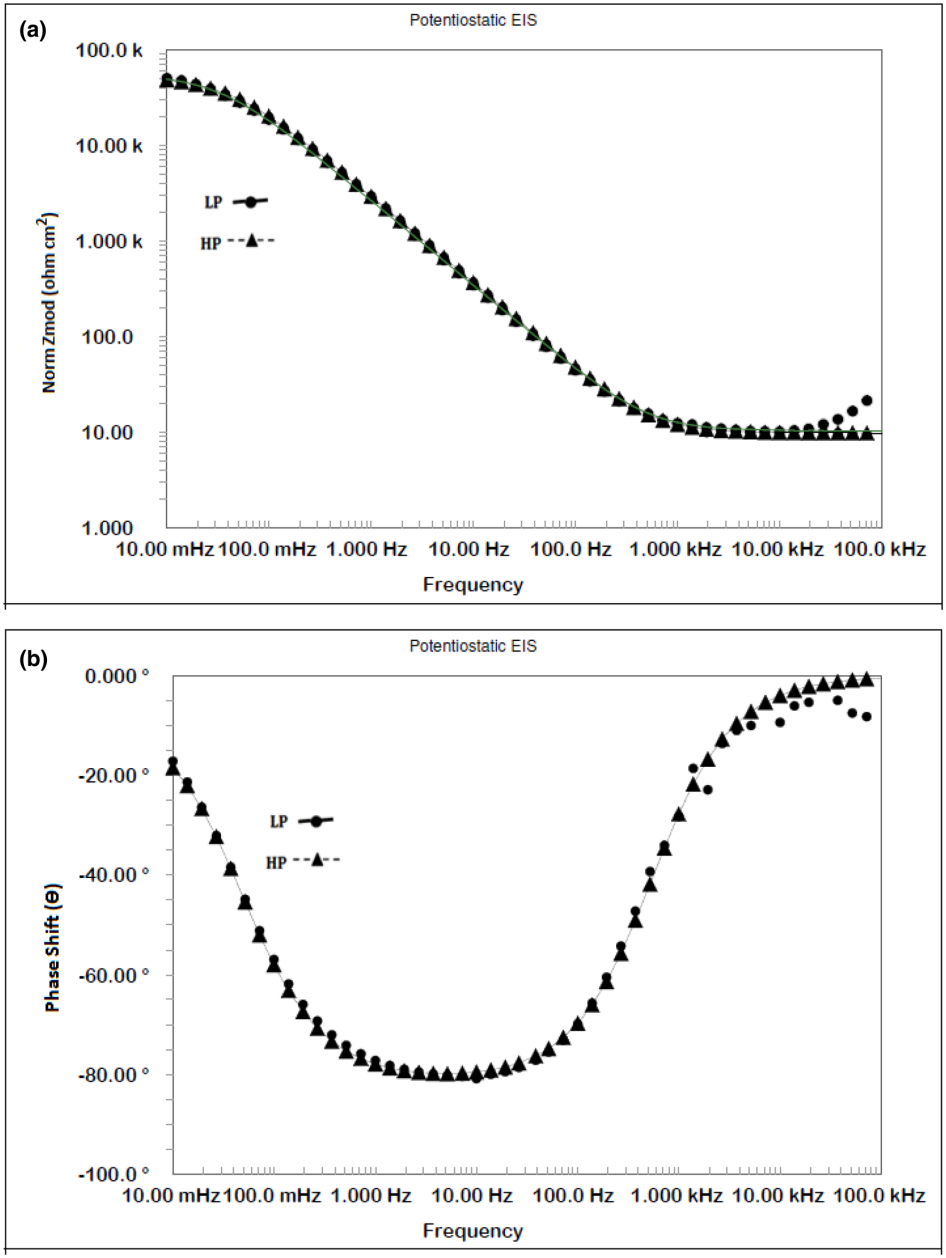
(**a**) Electrochemical frequency–impedance Bode plots for HP and LP steel bars after 24 h of their exposure in chloride added SPSL. (**b**) Electrochemical frequency–phase shift Bode plots for HP and LP steel bars after 24 h of their exposure in chloride added SPS.

**Figure 13:**
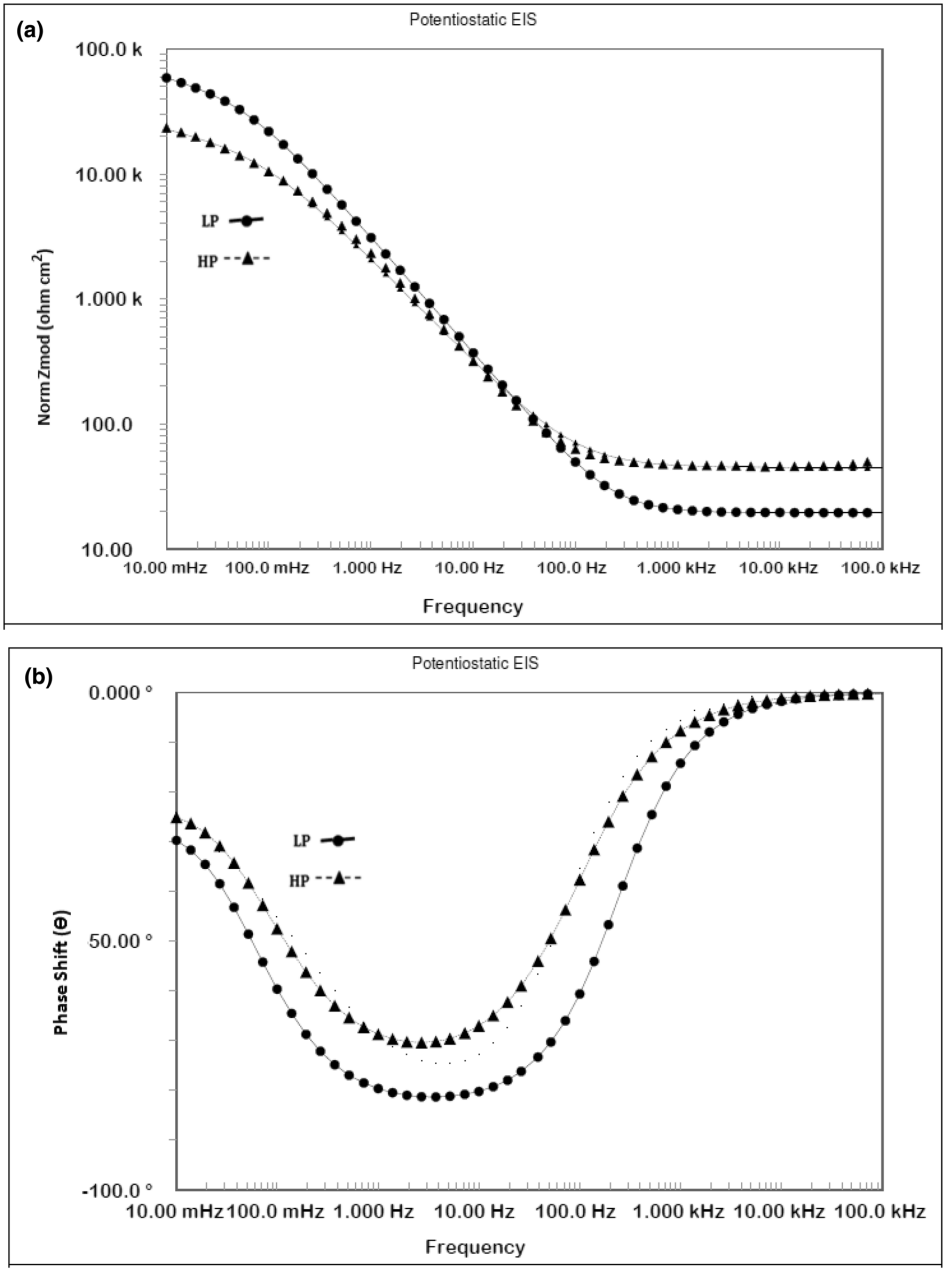
(**a**) Electrochemical frequency–impedance Bode plots for HP and LP steel bars after 192 h of their exposure in chloride added SPSL. (**b**) Electrochemical frequency–phase shift Bode plots for HP and LP steel bars after 192 h of their exposure in chloride added SPSL.

To assess the performance of the two steels in chloride added SPSL, they were tested after the addition of 0.6 M chloride (added as sodium chloride) to the solution. The results of EIS tests performed after 24 h of exposure in this test electrolyte are presented in Fig. 12a,b. As shown, the frequency–impedance curves the plots for both the steels overlap each other (Fig. 12a) at all the frequencies. The impedance at the lowest studied frequency (Z_max_), are significantly reduced compared with the corresponding tests performed in chloride-free SPSL (Fig. 10a). Both the steel appear to have a broad single maximum in the frequency–phase shift plots between 1 to 100 Hz (Fig. 12b) indicating that the corrosion reaction occurred with a single time constant. However, as discussed latter, in addition to the charge transfer, Warburg component was also associated with the corrosion reaction which was not visible in frequency-phase shift plots. The EIS studies were performed on the above samples kept exposed in the same chloride added SPSL for longer duration (192 h). The results are shown in Fig. 13a,b. Unlike the case of 24 h of exposure (Fig. 12a), the Z_max_ values at the lowest studied frequency for the two rebars after 192 h of exposure differ significantly from each other. Interestingly, the (Z_max_) for the LP rebar at 192 h of exposure remained almost the same as that at 24 h, whereas it deteriorated considerably for the HP rebar with longer durations of exposure. Another notable feature at higher frequencies (1 to 100 kHz) for both the rebars is observed. The impedance values are higher than that noted for 24 h exposure (Fig. 12a) with HP rebar bearing significantly greater values. The higher frequencies impedance is attributed to the resistance imparted by electrolyte and corrosion products formed on the surface of the test electrodes^47,48^. These results further indicate that the rusting caused by the chloride ions on the surface of HP rebar after longer durations of exposure was more pronounced than the LP rebar. In this case also, the corroding system appear to have a single maxima in intermediate frequency ranges (around 1 to 100 Hz) with phase shift Ɵ tending to 90°. However the experimental data also in this case could be fitted only after putting Warburg component in the simulated electrical circuit (discussed in the forthcoming paragraphs).

**Figure 14:**
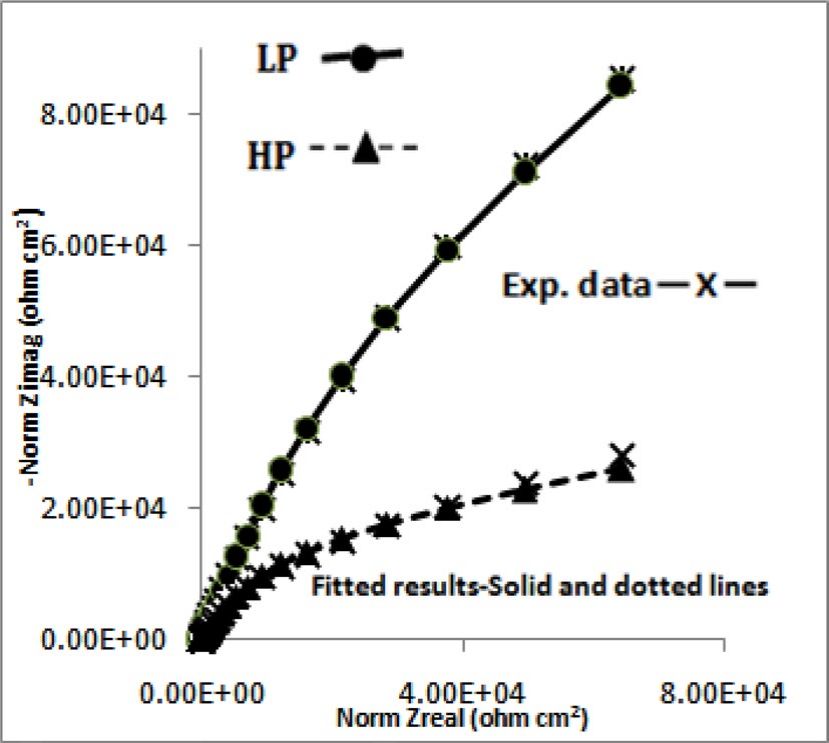
Electrochemical impedance plots in Nyquist form for HP and LP steel bars after 24 h of their exposure in chloride free SPSL.

**Figure 15:**
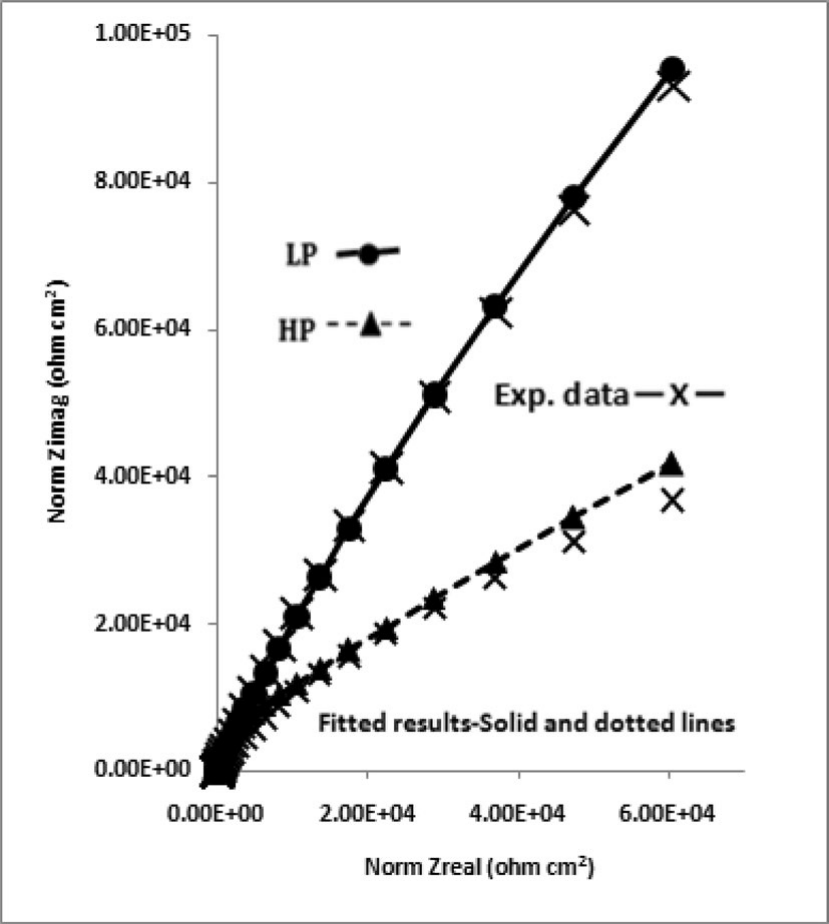
Electrochemical impedance plots in Nyquist form for HP and LP steel bars after 192 h of their exposure in chloride free SPSL.

**Figure 16:**
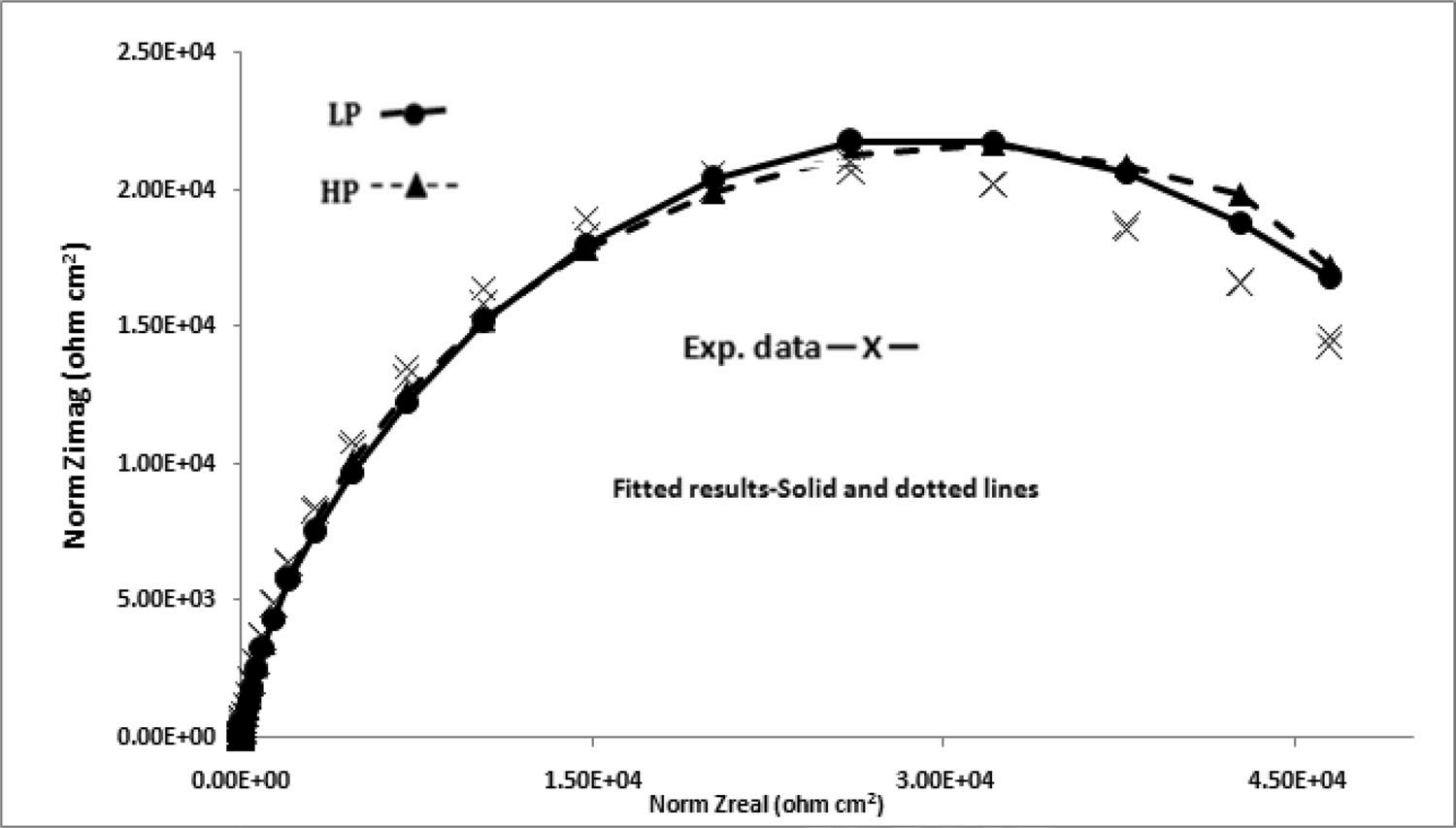
Electrochemical impedance plots in Nyquist form for HP and LP steel bars after 24 h of their exposure in chloride added SPSL.

**Figure 17:**
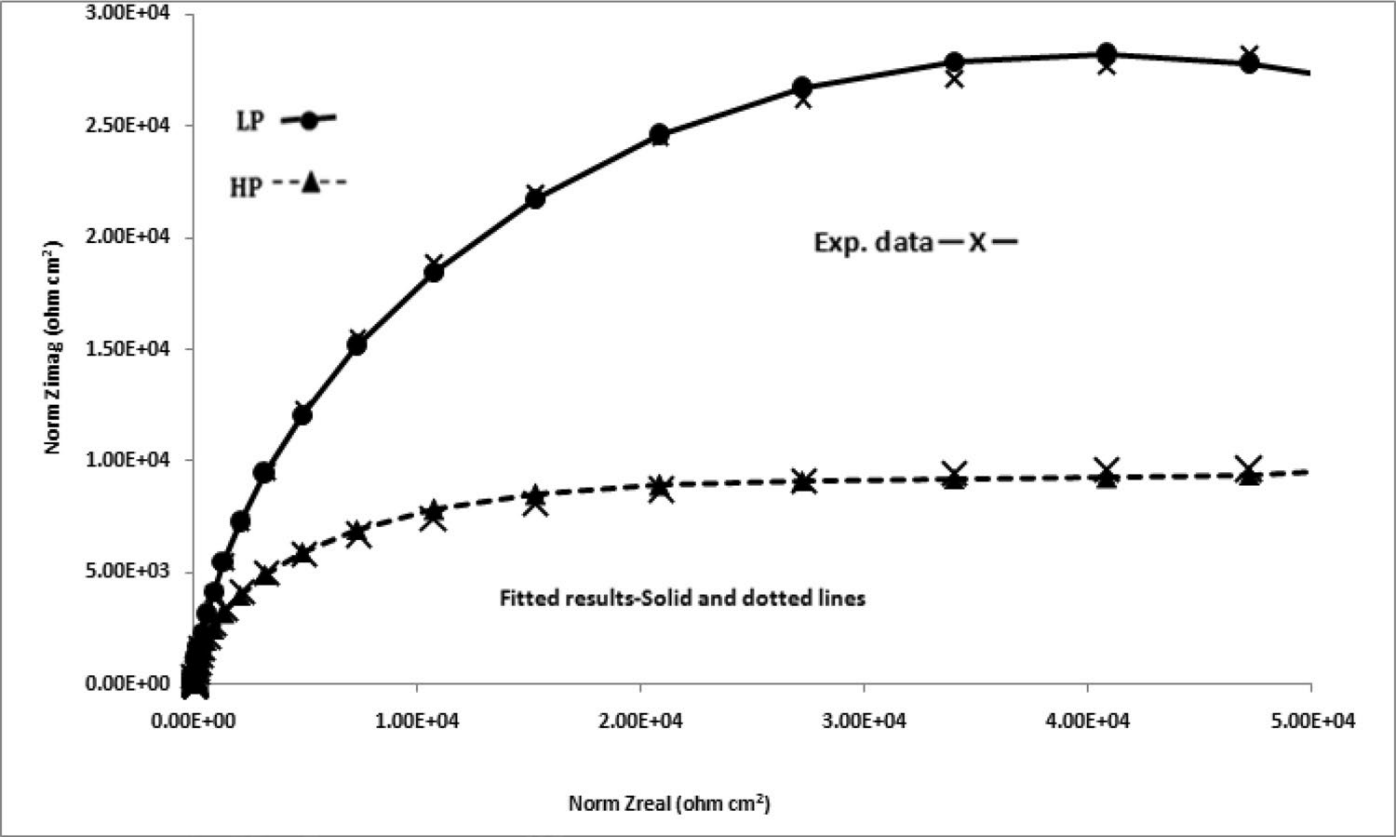
Electrochemical impedance plots in Nyquist form for HP and LP steel bars after 192 h of their exposure in chloride added SPSL.

The impedance data plotted in Nyqist form (real vs imaginary impedance) provide much important and valuable information about the corroding interface. In view of this the data of Figs. 10, 11, 12 and 13 were also plotted in this form and are presented in Figs. 14, 15, 16 and 17. It is seen that the features of Nyquist plots for rebars exposed in chloride free and chloride added pore solutions significantly differ. The plots for the first group of samples (exposed in chloride free SPSL) are closure to straight lines forming about 45^o^ angle between the abscissa and ordinate axes. The plots for the second set of samples exposed in chloride added solutions on the other hand tend to form depressed semi circles. These characteristics of the plots are associated with a spread of relaxation times and represented by substituting a constant phase element (CPE) in place of pure capacitor in the equivalent electrical circuit^47^. The CPEs (constant phase elements) which are defined by frequency independent phase angles are used for the parameterization of impedance values including non-ideal behavior of double layer, charge transfer resistance, ionic adsorption or diffusion across the corroding interface and uncompensated resistance(electrolytic ionic resistance (R_0_) between the interface of the solution and the electrode surface). Many researchers in the past have used such circuits to extract impedance data for rebars corroding in concrete pore solutions^49–51^. CPE is an empirically derived parameter and mathematically expressed as^52^:

3 $${\text{Z}} = \left( {{\text{j}}\omega } \right)^{ - \alpha } /{\text{Yo}}\_$$

**Figure 18:**
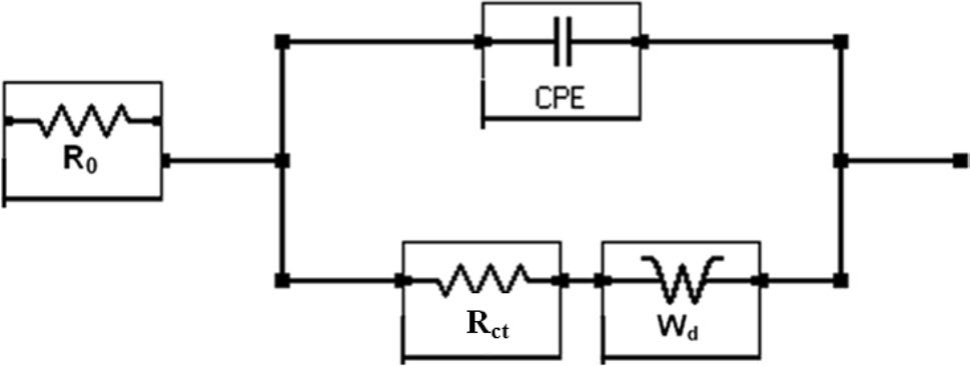
Equivalent electrical circuit of the CPE simplex model incorporating the CPE and the Warburg diffusion component (W); R_ct_ and R_s_ represent the charge transfer resistance and solution resistance, respectively.

Here Yo and α are respectively admittance modulus of an ideal capacitor and CPE factor. j is the imaginary unit and ω is angular frequency. Yo has unit Ω^−1^s^α^. The value of α is greater than zero but less than 1. The value of α = 1 for the interface corresponds to its behaviour as pure capacitor. In view of the non-ideal capacitance behaviour of the corroding interface of the two studied rebars (Figs. 14, 15, 16, 17) the impedance experimental data were fitted in different modelled circuits. Before fitting in simulated electrical circuits the experimental data were validated using Kramers-Kroning (KK) test. This method of validation is reported an effective way to see whether the experimental impedance data is characteristic of a linear and stable system and acceptable to proceed for further analysis^53,54^. It was noted that the fitting of the data with simple CPE elements in simulated circuit (incorporating un-compensated resistance (R_0_,), charge transfer resistance (R_ct_) and CPE element) did not yield the satisfactory fitting results. In view of this another component , the Warburg diffusion element (W_d_) was incorporated in this circuit, schematically shown in Fig. 18. This element referred as semi-infinite diffusion impedance presents mass transport hindrance experienced across the interface of the passive film on metal surface and the test electrolyte. More detail on development of this component at the corroding interface will be discussed under the forthcoming paragraphs. The extracted impedance data for the corroding rebars under different exposure conditions and test electrolytes are recorded in Table 2. It was observed that the best fitting of data with least error was noted with values of ‘α’ between 0.80 to 0.93. These values of ‘α’ indicate that the corroding interfaces neither behaved as ideal capacitor nor a pure resistor. As seen from the Figs. 14, 15, 16 and 17 the fits between the experimental and simulated data are reasonably good with a few simulated points especially at higher frequencies deviating from the experimental data.

**Table 2:** Quantitative data extracted from the EIS plots shown in Figs. 10, 11, 12 and 13 using the CPE model of the equivalent circuit presented in Fig. 18.

Steel rebars	Time (h)	R_0_ (Ω cm^2^)	R_ct_ (kΩ cm^2^)	W_d_ 10^−6^ × S × s^1/2^/cm^2^	Y_o_ (10^−6^ × S × s^α^/cm^2^)	α	Chi square (Χ^2^) × 10^–3^
**Test electrolyte: SPSL**							
LP	24	32	155.1	33.4	62.1	0.81	6.0
HP	24	26	121.0	142.5	63.9	0.87	21.5
LP	192	40	45.3	20.9	50.5	0.83	6.4
HP	192	41	4.8	60.7	53.6	0.93	9.2
**Test electrolyte: Chloride added SPSL**							
LP	24	9	46.7	726.3	64.3	0.90	4.3
HP	24	10	44.4	809.7	73.2	0.89	53.3
LP	192	19	46.5	153.5	57.8	0.93	23.7
HP	192	48	19.1	455.6	100.1	0.84	10.9

The results incorporated in this table show that the values of chi-square (Χ^2^) which is an indication of standard deviation in the results are of the order of 10^–3^. Some researchers opine that the values of chi-square for a good fitting should be below 10^–3^. Many others including Macdonald^55,56^, Ren et al.^57^ and Zhao et al.^58^ on the other hand suggest that the chi-square factor is a subjective value which sometimes leads to biased parameter estimates and merely 10^–3^ criteria should not be a deciding factor for the reliability of the data. Since the fitting of the data using the simulated equivalent circuit of Fig. 18 was good and Kramers–Kronig validity test with chi-square values in the range of 10^–5^–10^–7^ were recorded we proceeded to extract the fitting of data from EIS experimental results.

As stated above the Warburg diffusion (W_d_) is related to the impedance caused due to the diffusion of species from the solution-passive film interface to the passive film–metal interface^59,60^.

This element in equivalent electrical circuit is a contentious issue and is reported to account for diffusion (i.e., mass transfer, charged species etc.) processes, and is commonly used when the diffusion of species through the pores of the passive film or corrosion products controls the corrosion rate .The component is also related to ease of diffusion of species such as chloride and oxygen through the film formed at the interface^61,62^. The Warburg impedance (W_d_) does not provide any inkling whether it is due to the diffusion of oxygen, chloride or any other specie through the interface. The data for R_0_ (uncompensated resistance) recorded in Table 2 show that the values at lower duration of tests (24 h) for both the steels are comparable for the samples exposed in pure SCPSL as well as chloride added solutions. After longer durations, however the values are considerably higher than that noted for 24 h of tests, in both the solutions. As discussed, earlier R_0_ in addition to the solution resistance also incorporates the resistance caused by the surface film. At longer durations of exposure, the corrosion products formed on the surface of two steels added to the resistance. Further, as expected the chloride addition made the electrolyte more conducting which reduced the value of R_0_. A notable increase in R_0_ for the steels at 192 h of exposure in chloride added solution is attributed to the rust layer formed on their surface.

The values of charge transfer resistance (R_ct_) recorded in the above Table 2 are invariably low for HP than that for the LP steel rebars. These results suggest that LP develops more stable film than the HP steel exposed in SPSL both under chloride free as well as chloride added solutions. A significant difference between the LP and HP steel rebars is observed for the R_ct_ values recorded under all the test conditions. The addition of chloride to the test electrolyte significantly reduced the value for both the steels at shorter duration of exposure (in comparison to chloride free SCPL solution). After longer exposure (192 h) however, the values increased which is more pronounced for HP steel, it is attributed to the embedding of carbonate in rust layer making it more impervious. Since the rust was denser on HP steel the carbonate embedding effect was more visible than that on LP steel. The Warburg diffusion component (W_d_) recorded in the Table 2 are considerably higher for the steels exposed in chloride added solution. However no definite trend is noted especially when compared with the trends of R_0_ and R_ct_. Warburg impedance element (W_d_) depends on the concentration of the active material and diffusion coefficient of oxidant and reluctant species, number of mobile electrons, angular frequency and is expressed by the equation^63^:

4 $${\text{W}}_{{\text{d}}} = \frac{1}{\sqrt \omega }(1 + j)\left\{ {\frac{RT}{{\sqrt 2 n^{2} F^{2} }}\left[ {\frac{1}{{\sqrt {D_{0} } C_{0} }} + \frac{1}{{\sqrt {D_{R} } C_{R} }}} \right]} \right\}$$

In the above equation, D_O_ and D_R_ are diffusion coefficient of oxidant and reluctant, C_O_ and C_R_ represent the bulk concentrations of diffusing species, n is the number of electrons transferred, F is Faraday constant and ω stands for the radial frequency. As evident from the above equation the value of Warburg diffusion element is inversely related to diffusion coefficient and concentration of oxidizing and reducing components and their decreased values would increase W_d_. Since the steels were exposed in two different type of solutions (pure SPSL and chloride added SPSL), it is expected that in SPSL oxygen is diffusing species and in other case (chloride added SPSL) it is chloride ion that diffuse through the surface film. The diffusion coefficient of chloride is reported to have considerably lower than that of oxygen in concrete environments (the diffusion coefficient of chloride is reported of the order of 10^–12^ m^2^/s^64,65^ and that of oxygen is 10^–8^ m^2^/s^66^. About four order lower value of diffusion coefficient of chloride than that of oxygen justifies the higher magnitude of W_d_ for chloride added SCPL solution. In the case of mortar specimen, the warburg element was perhaps due to oxygen diffusion during the first cycle. However, in the 10th cycle it was most probably dominated by the chloride diffusion.

The Y_0_ values in Table 2 are the admittance i.e., the ease of reaction of the corroding interface. This values under all the test conditions are lower for LP than the HP steel indicating that the latter is more prone to the corrosive attack than the former steel (LP).

Another important parameter for the corroding interfaces is double layer capacitance (C_dl_) which is formed due to the existence of the electrical double layer (caused due to charge alignments) across the interface between the exposed metal and the corroding electrolyte. An increased value of C_dl_ is normally attributed to increase in surface area and roughness of the test electrode caused due to the progress of corrosion. The C_dl_ values for the corroding interfaces were computed from the extracted data of EIS plots, using the following equation^67^:

5 $${\text{C}}_{{{\text{dl}}}} = \left( {{\text{Y}}_{{\text{o}}} } \right)^{{{1}/\upalpha }} \left( {{1}/{\text{R}}_{{\text{o}}} } \right)^{{\left( {\upalpha - {1}} \right)/\upalpha }}$$

**Figure19:**
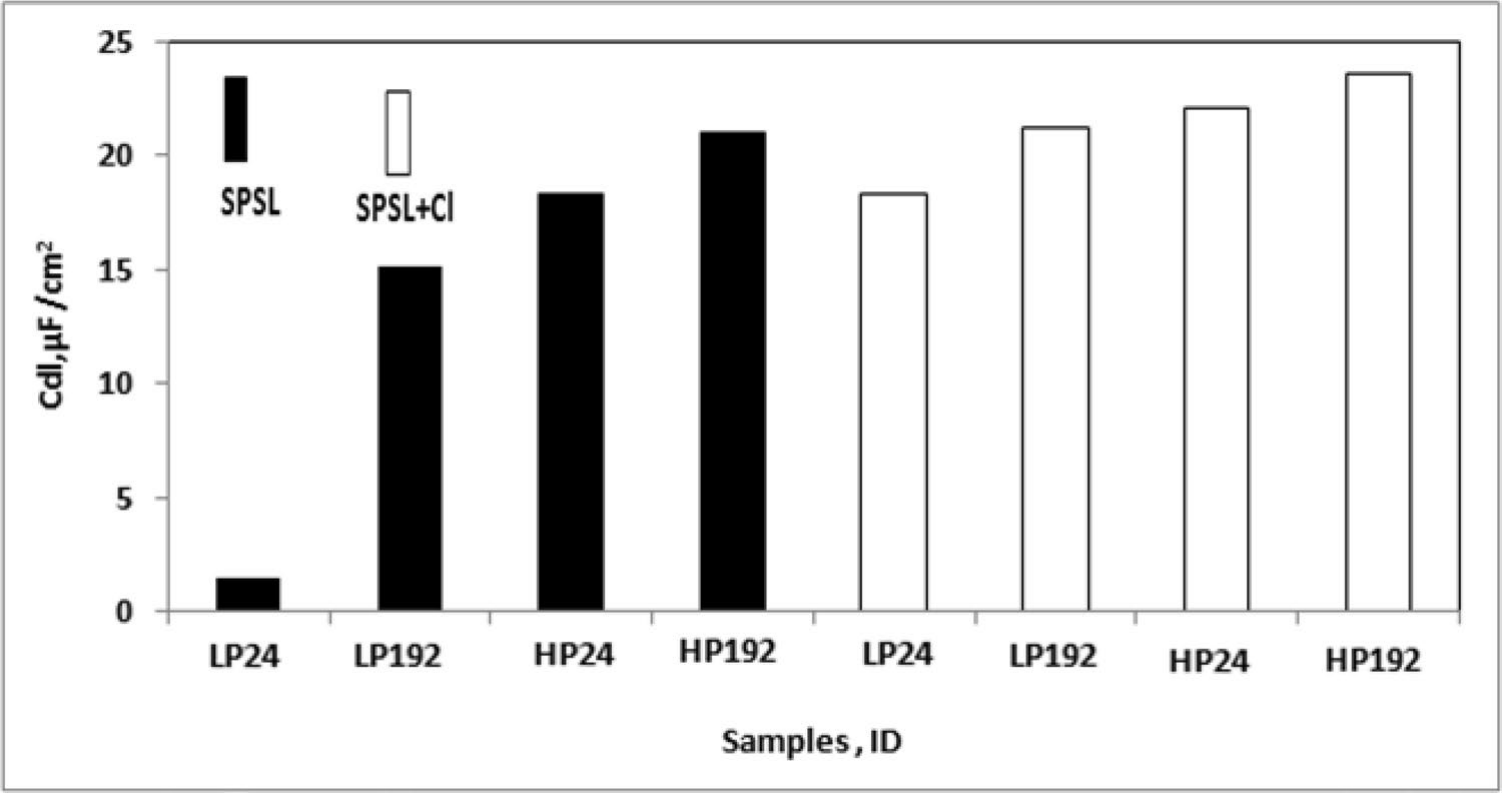
C_dl_ values calculated from EIS extracted data for LP and HP steel bars corroding under different exposure conditions.

The computed C_dl_ values for the two rebars exposed under different conditions are presented as column charts in Fig. 19. It is seen from the figure that a considerable difference in C_dl_ measured for LP and HP steel values are noted especially in chloride free SPSL. The values for LP are invariably lower than those recorded for HP steel rebar for shorter and longer duration of exposure. Many factors such as change in porosity of the film formed at the corroding interface, roughness and thickness are reported to influence the C_dl_ values^68–70^. It appears that in chloride free SPSL the lower values of C_dl_ for LP are due to a tortuous film formed on its surface vis-à-vis HP steel. This defect free passive film on LP effectively controlled the diffusion of oxygen through it towards the steel surface. The values of the C_dl_ of the two steels exposed in chloride added SPSL steels however did not exhibit very distinct differences. This may be attributed to thicker rust formed on both the steel surfaces under this condition of exposure.

### EIS studies of rebars embedded in mortars

The corrosion resistance performance of rebars exposed in mortar is more relevant to real life conditions. EIS tests were performed for the LP and HP steel rebars after they were embedded in mortar. The tests as described in the experimental details of the paper were continued for 10 cycles of wet/dry treatments (10 months).

**Figure 20:**
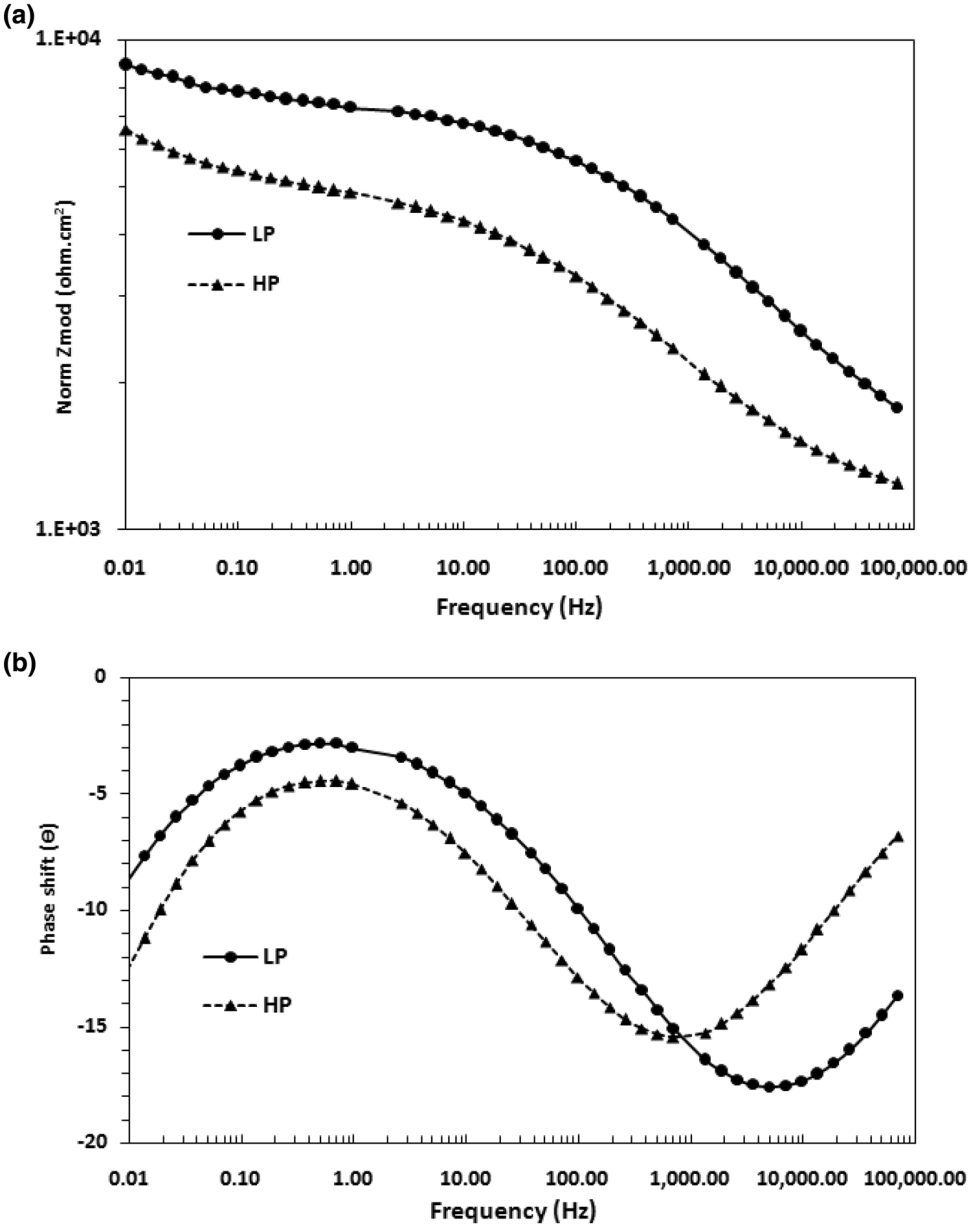
(**a**) Bode log frequency–log impedance plots for LP and HP steel rebars embedded in mortars after 1st cycle of wet/dry treatment in chloride solution. (**b**) Bode log frequency–phase shift plots for LP and HP steel rebars embedded in mortars after 1^st^ cycle of wet/dry treatment in chloride solution.

**Figure 21:**
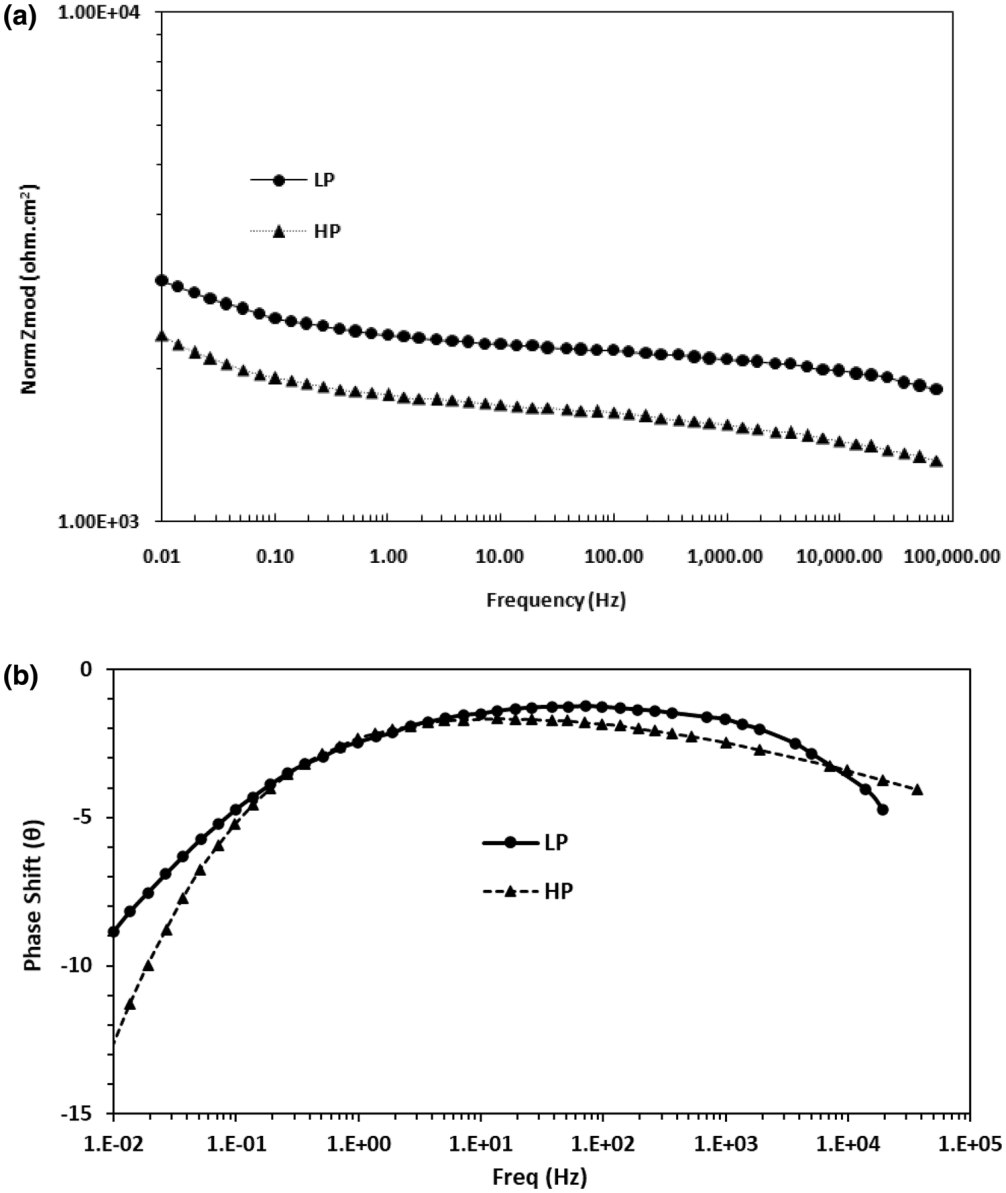
(**a**) Bode log frequency–log impedance plots for LP and HP steel rebars embedded in mortars after 10th cycle of wet/dry treatment in chloride solution. (**b**) Bode log frequency–phase shift plots for LP and HP steel rebars embedded in mortars after 10th cycle of wet/dry treatment in chloride solution.

The plots for the initial exposure durations (Cycle 1) and longer durations (after 10 cycles) are presented in Figs. 20 and 21, respectively. As shown, for both the periods of exposure, the impedance (Z_max_) at the lowest studied frequency (0.01 Hz) for the LP rebar was higher than that for the HP steel rebar (Figs. 20a and 21a). For the test conducted after one cycle of wet/dry treatment, two maxima were recorded in the frequency–phase shift plots for both the steels (Fig. 20b). After longer test durations (Cycle 10), only one maxima is visible in the frequency range of 10–100 Hz (Fig. 21b) but fitting of the experimental data was good only after Warburg diffusion element was added in the simulated electrical circuit.

These data plotted in Nyquist form exhibited a similar behaviour as recorded for SPSL solution i.e., forming straight line with about 45° angle between real and imaginary impedance axes (plots not showed).In view of this, the simulated equivalent electrical circuit as schematically presented in Fig. 18 was used to extract the quantitative impedance data also for these tests. The fitting of the data for shorter duration of exposures were satisfactory. For longer duration of exposure (10 cycles) distortion in fittings were recorded in higher frequency regions. Such distortions at high frequencies are attributed to inductive effects caused by stray capacitances caused due to the geometry of the mortars and electrochemical cell^69^. To minimize this effect the experiments were conducted keeping mortars in metal shield and spectra with minimum distortions were noted.The values for different components associated with reactions occurring at the interface of steel reinforcement bars embedded in mortars are presented in Table 3. The values of un-compensated resistance (R_0_) after ten cycles of wet/dry treatments are noted to decrease vis-a-vis shorter duration test. This is attributed to the diffusion and accumulation of chloride in the concrete pore solution after longer durations of the exposure.

**Table 3:** Quantitative data extracted from the experimental EIS data for the rebars embedded in mortars using CPE model of the equivalent circuit presented in Fig. 18.

Steel rebars	Test cycles	R_o_ (kΩ cm^2^)	R_ct_ (kΩ cm^2^)	W_d_ 10^−3^ × S × s^1/2^/cm^2^	Y_o_ (10^−6^ × S × s^α^/cm^2^)	*α*	Chi square (Χ^2^) × 10^–3^
LP	1	1.4	6.51	2.09	4.89	0.42	7.2
HP	1	1.3	4.08	2.00	13.0	0.43	16.3
LP	10	1.13	2.54	5.00	48.1	0.12	5.5
HP	10	1.05	1.95	5.41	72.9	0.12	11.9

The charge transfer resistance for both the mortars embedded rebars was reduced after longer durations (10 cycles) of wet/dry treatments. An opposite trend is noted for Y_0_ (admittance) indicating an increased vulnerability of both the rebars with longer durations of wet/dry treatments. Under all the test conditions R_ct_ values are higher and Y_0_ lower for LP than the HP steel, confirming the results of the other tests where LP steel rebar had an edge over the HP rebar. The increased W_d_ noted for the longer exposure (10 cycles) in comparison to shorter duration (2 cycles) test is probably due to lower diffusion coefficient of chloride (after 10 cycles, chloride is expected a dominant diffusion specie) than oxygen which was dominant at shorter exposure test. Under all the test conditions the performance of the LP steel rebar is clearly better than that of the HP rebar also in mortar-embedded conditions.

## Discussion

Phosphorus content in metals, alloys and coatings play very important role on corrosion, film formation, catalytic activity on reactions, microstructures and mechanical properties^72–75^. The foregoing test results for the LP and HP rebars indicate that increasing the P content in steel rebars has deleterious effects on their resistance to corrosion in alkaline concrete pore solutions. This finding is contrary to the reported beneficial effects of higher contents of P in low-alloy steels exposed to dry industrial environments^1,2,71–76^.

This contrast behaviour suggests that the reaction mechanism for the corrosion of steels with a high P content in an alkaline concrete pore solution differs from that in atmospheric environments. The corrosion resistance of metals is controlled by the protective nature of the passive film formed on their surfaces in a particular environment. The nature of the film is determined by the compositions of the test electrolytes; the bulk chemistry of the metals; the grain size of the metals; and the microstructure, non-metallic inclusions, and stability of the grain boundaries. The beneficial effect of P with Cu on the corrosion resistance of steels in industrial dry environment is attributed to the oxidation of P atoms of steel into phosphates by Cu ions and their co-precipitation with rust during the process of corrosion^77^. Phosphates are normally alkaline in nature, act as anodic inhibitor and facilitate the formation of a passive film on the steel surface^78^. This assertion is acceptable for corroding electrolytes where the moisture/water content at the corroding interface is low, such as steels exposed in an industrial dry atmosphere. However, it may not be valid for steels exposed in aqueous solutions. In solutions such as SPSL and mortars having plenty availability of moisture / water, the formed phosphate concentration is probably too low to impart passivation effects on the steel surface. It is to be noted that phosphate anions in concrete pore solution act as an anodic corrosion inhibitor^78,79^.The performance of such inhibitors is sensitive to their concentration in the test electrolytes and accelerate corrosion if present below a threshold concentration. In rebars steels the addition of P is maintained in the range of 0.06–0.08% to avoid other deleterious effects. This low concentration of P is inadequate to meet the threshold concentration for the passivation of steel surface. This argument gets support from the findings in reference^80^ where very high level of the addition of P (0.5% and above) in steel provided good protection to the surface of steel rebars exposed in simulated concrete pore solution.

In addition to the above mechanism the increased corrosion rate of HP steel appears to be associated with the inherent tendency of P to segregate in ferrite grain boundaries of steels causing accelerated ionisation of Fe atoms from the lattice. Alloying of P in steels affects their properties in different ways depending on the concentration. A high concentration of P in Fe can cause segregation at ferrite grain boundaries leading to their reduced cohesion, whereas a lower content leads to the formation of a solid solution with Fe without grain boundary segregation^79^. A bulk concentration of 0.013% of P in steels was reported to reduce the ferrite grain boundary cohesive energy by 10%^82^. An increased concentration of P in steels is known to have adverse effects on the integrity of the grain boundaries^82^. The cohesive energy of a grain boundary is defined as the energy needed to separate the two adjacent grains to form free surfaces. The bulk concentration of P in the HP steel rebar is significantly higher (0.064%) than the aforementioned reported value (0.013%)^82^. Theoretical calculations and experimental evidence suggest that the electronic charge transfer between the segregated atoms and the host metal atoms was responsible for this loss of cohesiveness of the grain boundaries^81^. The segregated metal atoms (here, P) are more electronegative than the host atoms (Fe). The charge transfer from the latter atoms to the former atoms reduces the number of electrons that can participate in metal–metal bonds that hold the grain boundaries together. Abundant experimental evidences indicate that grain boundaries with poor cohesion cause different types of metallic failures, including intergranular corrosion and stress-assisted corrosion^83–87^. The higher rate of corrosion of HP steel rebars in concrete environments may be attributed to the P present in a concentration range causing segregation and reduction in the cohesiveness of ferrite grain boundaries and ultimately leading to accelerated rate of corrosion. However, this degradation effect is absent in LP steel owing to the lower P content.

**Figure 22:**
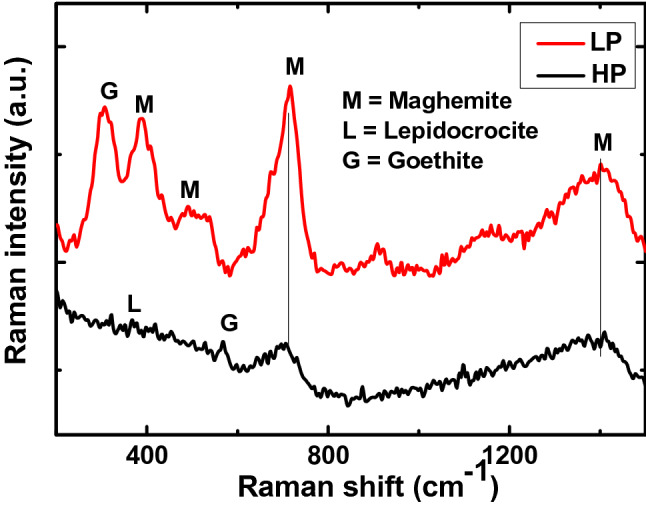
Raman spectra of rust formed on steel rebars embedded in mortar after 10 cycles of wet/dry treatment in chloride solution.

The nature of corrosion products formed on meals and alloys surfaces exposed in any environment provide very convincing and strong clues about the corrosion characteristics of the studied materials. The formation of thermodynamically stable corrosion products on metals surface provides improved protection than those for unstable phases. The intensity of peaks studied by Raman spectroscopy for stable phases of maghemite and goethite formed on the surface of LP steel bars are reasonably strong as seen from Fig. 22. On the surface of the HP steel, weak peaks corresponding to lepidocrocite (metastable oxide of iron) and goethite are present (Fig. 22). Compounds of P are reported to affect the nature of corrosion products on steel surface^12^. The maghemite and goethite phases of Fe rust present on the surface of LP steel rebars are thermodynamically more stable than the lepidocrocite phase of rust present on the surface of HP steel bars. Astable phase of rust is strongly bonded to the steel surface, has sound morphologies, and provides a tortuous path for the diffusion of moisture, oxygen and chloride ions at the metal surface It appears that the higher content of phosphorus in HP discouraged the transformation of thermodynamically weaker lepidocrocite in to stable forms of maghemite and goethite phases of rust. The leached out P from HP which transforms into phosphide and phosphene in alkaline environments^35,36^ probably impeded the transformation of lepidocrocite in to maghemite and goethite. This is probably the reason why the LP steel rebar had a lower rate of corrosion during the wet/dry cycle than the HP steel rebars (Figs. 3, 4).

## Conclusions

Content of phosphorus in rebars steel considerably affects the nature of film formed at the interface of steel–concrete pore solution . A higher content of P (0.064%) has deleterious effect on the stability of the steel-pore solution interface vis-a-vis lower P content (< 0.016%).The localized corrosion in higher P content steel (HP)is significantly high than the normal P containing steel (LP).Cyclic polarization indicates that in the presence of chloride ions (0.6 M) the passive film formed on the surface of both the steels is deteriorated leading to lowering of break down potential of HP than the LP steel. However, in absence of chloride ions both the steels were immune to localized corrosion. EIS studies also corroborate the findings of weight loss and polarization techniques confirming that the HP steel was more vulnerable to corrosion than the LP steel rebars. The diffusion coefficient for Cl and O_2_ through the film formed on the surface of LP steel was smaller than that noted for HP steel rebars. The double layer capacitance calculated from the admittance, solution resistance and constant phase element factor provided higher values for the HP in comparison to the LP rebars. It was attributed to the higher rate of corrosion attack on HP steel resulting in increased surface area of this rebar. The accelerated rate of corrosion of HP than LP steel bars appears due to leaching out of P from the matrices of the former stated steel, formed phosphene reacting with alkaline solution which facilitated the oxygen evolution reaction of the corrosion. Raman spectroscopic analysis of corrosion products formed on the surface of the studied steels suggest that the higher content of P in HP steel discouraged the transformation of unstable lepidocrocite phase of rust in to maghemite and goethite.
